# *Dullard/Ctdnep1* Modulates WNT Signalling Activity for the Formation of Primordial Germ Cells in the Mouse Embryo

**DOI:** 10.1371/journal.pone.0057428

**Published:** 2013-03-04

**Authors:** Satomi S. Tanaka, Akihiro Nakane, Yasuka L. Yamaguchi, Takeshi Terabayashi, Takaya Abe, Kazuki Nakao, Makoto Asashima, Kirsten A. Steiner, Patrick P. L. Tam, Ryuichi Nishinakamura

**Affiliations:** 1 Department of Kidney Development, Institute of Molecular Embryology and Genetics (IMEG), Kumamoto University, Kumamoto, Japan; 2 Laboratory for Animal Resources and Genetic Engineering, RIKEN Center for Developmental Biology (CDB), Kobe, Japan; 3 Research Center for Stem Cell Engineering (SCRC), National Institute of Advanced Industrial Science and Technology (AIST), Tsukuba, Japan; 4 Embryology Unit, Children’s Medical Research Institute, New South Wales, Australia; 5 Sydney Medical School, University of Sydney, New South Wales, Australia; National Cancer Center, Japan

## Abstract

Dullard/Ctdnep1 is a member of the serine/threonine phosphatase family of the C-terminal domain of eukaryotic RNA polymerase II. Embryos lacking Dullard activity fail to form primordial germ cells (PGCs). In the mouse, the formation of PGCs is influenced by BMP4 and WNT3 activity. Although Dullard is reputed to negatively regulate BMP receptor function, in this study we found mutations in *Dullard* had no detectable effect on BMP4 and p-Smad activity. Furthermore *Dullard* mutations did not influence the dosage-dependent inductive effect of *Bmp4* in PGC formation. However, *Dullard* may function as a positive regulator of WNT signalling. Combined loss of one copy each of *Dullard* and *Wnt3* had a synergistic effect on the reduction of PGC numbers in the compound heterozygous embryo. In addition, loss of Dullard function was accompanied by down-regulation of WNT/β-catenin signalling activity and a reduction in the level of Dishevelled 2 (Dvl2). Therefore, Dullard may play a role in the fine-tuning of WNT signalling activity by modulating the expression of ligands/antagonists and the availability of Dvl2 protein during specification of the germ cell lineage.

## Introduction

Specification of the germ cell lineage in the mouse begins with activation of *Prdm1* (*Blimp1*) in a subset of epiblast cells in the proximal region of the pre-gastrulation mouse embryo [1]. Analysis of germ cell formation in mutant mouse embryos has revealed the critical role of bone morphogenetic protein (BMP) signalling in the induction of primordial germ cell (PGC) precursors. The PGC population is lost or greatly reduced in embryos that are deficient for BMP activity, which is caused by losses of *Bmp4* and *Bmp8b* in the extraembryonic ectoderm, and *Bmp2* and *Alk2* (encoding a Type I BMP receptor) in the visceral endoderm, or *Smad1, 4* and *5* (signal transduction factors) in the embryo [2]–[12]. In addition, WNT signalling plays a role in PGC formation. PGCs are absent in embryos lacking *Wnt3* activity and WNT3A is required for priming epiblast cells to respond to induction by BMP4 to differentiate into PGCs [13].

*Dullard* (also known as *C-terminal domain nuclear envelope phosphatase 1; Ctdnep1*) was identified as a gene that is expressed in the pronephros and neural tissues of *Xenopus laevis* embryos [14]. *Dullard* encodes a protein serine/threonine phosphatase with a characteristic catalytic motif, DXDX(T/V). It is a member of an emerging family of phosphatases that dephosphorylate target substrates [15], [16]. This family is also known as the phosphatase family of the C-terminal domain (CTD) of eukaryotic RNA polymerase II (polII), which dynamically regulates transcription by recruiting different factors to mRNA through its multiple phosphorylation activities [17]. Other SCP/transcription factor IIF-interacting CTD phosphatases that are closely related to Dullard, e.g. small CTD phosphatases (SCPs), play a role in modulation of the expression level of specific genes. Such phosphatases silence neuronal genes in non-neuronal cells to suppress inappropriate neuronal gene expression during cell fate decision. This regulatory activity is mediated through an interaction with the repressor element 1-silencing transcription factor/neuron-restrictive silencer factor (REST/NRSF) complex [18]. Bioinformatic analyses of human DULLARD has revealed that the protein contains two potential membrane-spanning regions in the N-terminal, which may direct the localization of DULLARD to the nuclear envelope, where it dephosphorylates a nuclear membrane-associated phosphatidic acid phosphatase in human cell line cells [16]. Immunostaining further revealed punctuate localization of DULLARD in the nucleus and cytoplasm [16], suggesting that Dullard might have other target substrates that are not only associated with the nuclear envelope. Indeed, Dullard forms a protein complex with the BMP Type II receptor to promote its degradation [15], and suppression of BMP signalling may underlie its induction of neural tissue formation in the *Xenopus* embryo [15]. Dullard also interacts with BMP Type I receptors to repress their BMP-dependent phosphorylation. Therefore, Dullard might regulate the level of BMP signalling via its phosphatase activity to dephosphorylate BMP receptors, leading to their degradation [15]. Given the essential function of BMP signalling in the induction of PGCs, it is anticipated that factors modulating BMP signalling activity, such as Dullard, will affect germ cell specification.

In this study, we have demonstrated a critical requirement for Dullard in the formation of PGCs in the mouse embryo. However, results of the genetic study showed that loss of *Dullard* has no discernible effect on the expression of *Bmp4* and Smads, and does not affect the dose-dependent inductive activity of BMP4 on PGC formation. Instead, Dullard functions as an agonist that modulates WNT signalling activity to facilitate the formation of PGC precursors.

## Materials and Methods

### Ethics Statement

All animal experiments were approved by the Animal Care and Use Committee of Kumamoto University (#A24-110). Protocols were performed in accordance with the NIH Guide for the Care and Use of Laboratory Animals.

### Generation of *Dullard* Mutant and Compound Mutant Mice

Two types of embryonic stem (ES) cells harbouring modified *Dullard* alleles (*Dullard^+/−^* and *Dullard^+/LacZ^*) were generated by gene targeting (Fig. S1). Mutant mouse embryos generated from *Dullard^+/−^* or *Dullard^+/LacZ^* ES cells displayed similar phenotypes, and were therefore designated collectively as *Dullard^−/−^* embryos in this study. Expression of *Dullard* was reported by *LacZ* expression in *Dullard^+/LacZ^* embryos. For genetic interaction studies, *Dullard^+/−^* mice were crossed with *Bmp4^+/−^* mice (C57BL/6×CBA) or *Wnt3^+/−^* mice (C57BL/6) to produce compound mutant embryos. *Dullard^+/LacZ^* mice are available from RIKEN CDB (Acc. No. CDB0474K; http://www.cdb.riken.jp/arg/mutant%20mice%20list.html). *Dullard* ^+/−^ mice have been deposited at the Center for Animal Resources and Development (CARD), Kumamoto University (Acc. No. 466).

### Expression Vector Construction and Subcellular Localization of the Dullard Fusion Protein

An expression vector containing Dullard-enhanced green fluorescent protein (EGFP; fused to the C-terminal end of Dullard) under the control of the *CMV* promoter (*pEGFP-N1,* Clontech Laboratories, Inc., Mountain View, CA, USA) was transfected into NIH-3T3 cells. Then, the localization of Dullard-EGFP signals in the endoplasmic reticulum (ER) was visualized by anti-calreticulin immunostaining of the ER (Stressgen Bioreagents, Victoria, BC, Canada). Similar experiments were performed using a Dullard-FLAG fusion protein expression vector. Confocal imaging was carried out under a Leica SP2 confocal scanning system (Leica Camera AG, Wetzlar, Germany).

### In situ Hybridization, Immunostaining and Histochemical Staining of Embryos

Procedures for whole mount and section in situ hybridization, immunostaining, detection of alkaline phosphatase (AP) activity, and detection of β-galactosidase activity by X-gal staining have been described elsewhere [19], [20]. A cDNA fragment of *Dullard* (775 bp) spanning the open reading frame was used as the riboprobe. Other probes were: *Cer1* (from R. R. Behringer), *Lhx1* (*Lim1*), *Brachyury* (*T*) and *Bmp4* (from J. Rossant), *Fgf8* [21], *Eomes* [22], *Flk1/Vegfr2* [23], *Id1* [24], *Id2* [25], and *Wnt3* [26]. Antibodies used were: anti-Dppa3 (1∶1000; Pgc7/Stella, [27]), anti-Ifitm1 (1∶1000; mil-2/Fragilis2), anti-Ifitm3 (1∶1000; mil-1/Fragilis, [28]), anti-WNT3/3a (1∶50; Santa Cruz Biotechnology, Santa Cruz, CA, USA), anti-Axin2 (1∶50; Abcam plc, Cambridge, UK), anti-active β-catenin (ABC, 1∶50; Upstate Biotechnology), anti-phosphorylated Smads (p-Smads, 1∶100; p-Smad1/p-Smad5/p-Smad8; Cell Signaling Technology, Inc., Beverley, MA, USA), and Alexa Fluor 488- and Alexa Fluor 594-conjugated secondary antibodies (1∶750; Molecular Probes, Eugene, OR, USA). Fluorescence imaging was performed under an Olympus IX71 microscope and DP71 capturing system (Olympus, Tokyo, Japan). Two to three specimens were analysed for the expression of each gene or marker.

### RNA Extraction and Real-time Quantitative (Q)-PCR

Total RNA was extracted using RNeasy Plus Micro RNA extraction kits (Qiagen, Hilden, Germany). Reverse transcription was carried out with 0.2 µg RNA using a ReverTra Ace qPCR RT Master mix with gDNA Remover (Toyobo, Osaka, Japan). For Q-PCR, a Thermal Cycler Dice TP800 (Takara Bio Inc., Shiga, Japan) and Thunderbird SYBR-green qPCR mix (Toyobo) were used. The standard curve method was used to determine the relative quantities of mRNA, and expression levels were normalized to those of *Gapdh* (internal control). Two to three independent samples were analyzed as technical duplicates. Amplification of was performed with primer sets described in the related publications: *Brachyury*, *Ifitm3, Dppa3* and *Arbp* [29], *Wnt3* [30], *Axin2* [31] and *Goosecoid* [32]. Primers for *Dullard, Bmp4, Lef1, Dkk1, Id1, Msx2, Pou5f1* and *Gapdh* were purchased from Takara Bio Inc. Primers for *Ifitm1* amplification were: 3′- acatgcctgagatctccacg-5′ and 3′- atggtgaagaacagggagc-5′.

### Microarray Analysis

Microarray analysis was performed according to the Agilent Expression Array Analysis manual (Agilent Technologies, Palo Alto, CA, USA). cDNA was amplified from 20 ng total RNA using Ovation Pico WTA system V2 (NuGEN Technologies, San Carlos, CA, USA). Cy3-labelled cDNA was hybridised to a Whole Mouse Genome 4×44K v2 array (Agilent Technologies). Data normalisation and analyses were performed using the Agilent Feature Extraction program (Agilent Technologies). When the gene had multiple signal data, the average was indicated as a fold-change. The data have been deposited in the Gene Expression Omnibus database under the accession number 16596277.

### Western Blotting and Glutathione S-transferase (GST) Pull-Down Assay

For western blotting, the antibodies used were: anti-β-catenin (1∶2000; BD Transduction Laboratories, Franklin Lakes, NJ, USA), anti-ABC (1∶1000; Upstate Biotechnology), anti-Dvl2 (1∶1000; Cell Signaling Technology), anti-β-tubulin (1∶1000; Sigma-Aldrich) and an AP-conjugated secondary antibody (1∶10000; Promega, Madison, WI, USA). The relative amount of proteins was determined using the NIH ImageJ (ver. 1.44p) image processing program.

Construction of GST-Dullard expression vectors followed procedures described elsewhere [15], [16]. The GST-pull-down assay was performed in HEK293 cells transfected with Myc-tagged Dvl1, Dvl2 and Dvl3 expression vectors [33]. The results were collated from two independent experiments.

### Statistical Analyses

Statistical analyses were performed using the Student’s *t*-test, with p-values of <0.05 indicating statistically significant differences between datasets.

## Results

### *Dullard* Expression in Early Postimplantation Embryos and NIH-3T3 Cells

**Figure 1:**
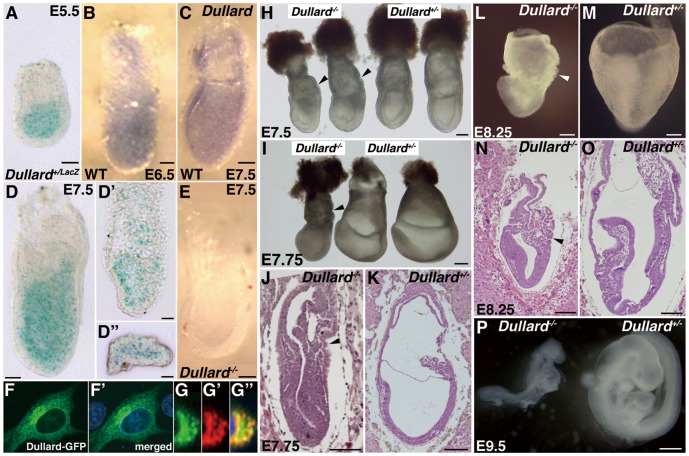
Dullard expression and mutant phenotype. (**A**) Embryonic day (E)5.5 *Dullard^+/LacZ^* embryo showing LacZ expression in the epiblast, but not in the visceral endoderm. (**B, C**) *Dullard* expression revealed by whole mount in situ hybridization of (**B**) E6.5 early streak-stage and (**C**) E7.5 early bud wild-type (WT) embryos. (**D**) E7.5 no bud-stage *Dullard^+/LacZ^* embryo, and (**D′**) sagittal and (**D′′**) transverse sections. (**E**) No *Dullard* expression in the E7.5 late bud-stage *Dullard^−/−^* embryo. (**F–G′′**) Localization of Dullard-enhanced green fluorescent protein (EGFP) fusion protein in transfected NIH-3T3 cells. (**F**) Dullard-EGFP (green), (**F′**) merged image with DAPI nuclear staining (blue). (**G**) Dullard-EGFP (green), and (**G′**) anti-calreticulin immunostaining (red) of the ER and (**G′′**) merged image with DAPI nuclear staining (blue). (**H**, **I**) Ruffled endoderm in the posterior of the yolk sac (arrowhead) of *Dullard^−/−^* embryos at (**H**) E7.5 late streak to early bud stage and (**I**) E7.75 late bud to head fold stage. (**J**) Sagittal sections of E7.75 *Dullard^−/−^* and (**K**) *Dullard^ +/−^* embryos. *Dullard^−/−^* embryos did not form the allantois or amniotic fold (arrowhead). (**L**) E8.25 *Dullard^−/−^* embryo showing retarded development compared with that of a (**M**) *Dullard^ +/−^* littermate. (**N**) Sagittal sections of an E8.25 *Dullard^−/−^* embryo showing poor development of extraembryonic structures (allantoic bud, yolk sac, and amnion; arrowheads) and the embryo proper compared with those in (**O**) *Dullard^ +/−^* embryos. (**P**) E9.5 *Dullard^−/−^* embryo (left) showing arrested development compared with that of a *Dullard^ +/−^* embryo (right). Scale bars = 100 µm (**A–E**, **H–O**) and 200 µm (**P**).

*Dullard* was expressed in the epiblast but not in the visceral endoderm of embryonic day (E)5.5 (*LacZ* expression, Fig. 1A) and E6.5 (Fig. 1B) embryos. In E7.5 embryos, *Dullard* was expressed in the ectoderm and mesoderm, but not in the definitive endoderm (Fig. 1C; *LacZ* expression, Fig. 1D–D′′). No *Dullard* mRNA was detected in E7.5 *Dullard^−/−^* embryos by in situ hybridization (Fig. 1E) or Q-PCR (Fig. S2A).

In NIH-3T3 cells that expressed Dullard-EGFP or Dullard-FLAG fusion protein, EGFP and FLAG signals were localized preferentially to the perinuclear domain and calreticulin-positive ER (Fig. 1F–G′′; FLAG data not shown). These results were consistent with the previously reported localization of endogenous Dullard and the transfected Dullard fusion proteins in the perinuclear domain of mammalian and yeast cells, respectively [16]. Dullard was also distributed in a punctuate pattern in the cytoplasm and nucleus (Fig. 1F, F′ and data not shown; [16]).

### Loss of Dullard Shows Little Effect on Embryonic Patterning but Affects the Morphogenesis of Extraembryonic Tissues

*Dullard^−/−^* embryos were present at the expected Mendelian ratio in E7.5 litters (23/108 = 21.3%) produced by intercrossing *Dullard^+/−^* mice. *Dullard^−/−^* embryos completed gastrulation and displayed proper anterior–posterior patterning. Various tissue markers were expressed appropriately in *Dullard^−/−^* embryos, namely *Cer1* in the anterior domain of visceral endoderm (Fig. S3A, B; [34]); *Lhx1* in the anterior domain of anterior mesendoderm and nascent mesoderm (Fig. S3C; [35]), as well as *Eomes* (Fig. S3D; [36]) and *Brachyury* (*T*, Fig. S3E–F′; [37]) in the primitive streak.

At E7.5–7.75, *Dullard^−/−^* embryos displayed ruffling of the yolk sac endoderm on the posterior side of the embryo (Fig. 1H, I) and a diminutive exocoelomic cavity (Fig. 1J, K). *Fgf8* expression was weaker in the posterior segment of the primitive streak (Fig. S3G, H; [21]). At E8.25, *Dullard^−/−^* embryos were clearly distinguished from *Dullard^ +/+^* and *Dullard^+/−^* littermates by poorly developed head folds and trunk, clumping of the extraembryonic mesoderm, a pleated yolk sac endoderm, and absence of the allantois and amnion (Fig. 1L–O, and Fig. S3L–W). In the E8.25 *Dullard^−/−^* embryo, the expression pattern of *Brachyury* in the primitive streak and nascent mesoderm (Fig. S3L) resembled that in the younger (E7.5) embryos (Fig. S3N) and not *Dullard^+/−^* littermates (Fig. S3M). In the extraembryonic mesoderm, *Flk1/Vegfr2* expression was reduced (Fig. S3I, J, O–Q; [23]), but the expression of *Id2* and *Bmp4* appeared to be unaffected (Fig. S3K, R–T). Only two (2/22 = 9.1%) very retarded *Dullard^−/−^* embryos with poor body development were recovered at E9.5 (Fig. 1P). No viable *Dullard^−/−^* embryos (0/25) were found by E11.5.

Microarray analysis of E7.5 *Dullard^+/−^* and *Dullard^−/−^* embryos revealed further defects in mesoderm development (Table S1). Anterior mesoderm markers, *Gsc* and *Lefty1,* were up-regulated in *Dullard^−/−^* embryos (6.9-fold and 4.5-fold, respectively), while nascent mesoderm markers *Brachyury* and *Fgf4* were down-regulated (2.3-fold and 3.1-fold, respectively). *Nkx1-2* (*Sax1*), which is expressed in the primitive streak, was down-regulated in *Dullard^−/−^* embryos (2.5-fold). Increased *Gsc* expression and reduced *Brachyury* and *Nkx1-2* expression were confirmed by Q-PCR (Fig. S4). Dullard activity is therefore not essential for embryonic patterning but is required for maintenance of the primitive streak and differentiation of the extraembryonic tissues.

### *Dullard^−/−^* Embryos are Depleted of PGCs

**Figure 2:**
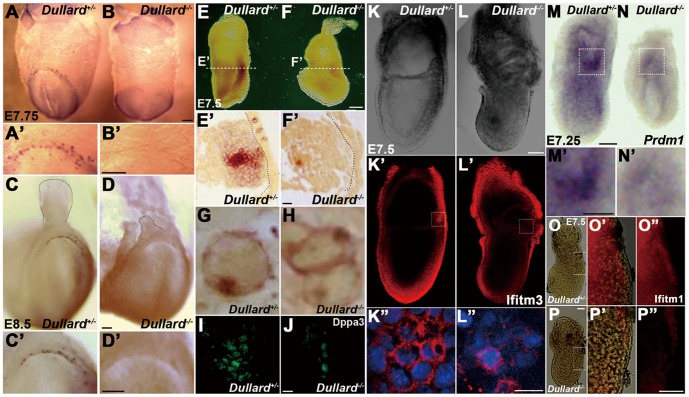
*Dullard^−/−^* embryos fail to form primordial germ cells (PGCs). (**A–H**) Absence of alkaline phosphatase (AP)-positive PGCs in *Dullard^−/−^* embryos at the (**A, B**) E7.75 neural plate stage (**A′**, **B′**; higher magnification views) and (**C–D**) E8.5 early somite stage (**C′**, **D′**; higher magnification views). (**E–H**) Histology of E7.5 (**E, E′**) *Dullard^ +/−^* and (**F, F′**) *Dullard^−/−^* embryos revealing the absence of AP-positive cells in the endoderm of *Dullard^−/−^* embryos, while few positive cells in the mesoderm displayed (**H**) the characteristic staining pattern (in the cell membrane and a cytoplasmic aggregate) of PGCs in the endoderm of (**G**) *Dullard^ +/−^* embryos. (**I, J**) Markedly reduced population of Dppa3-expressing cells in the E7.5 *Dullard^−/−^* embryo (confocal images). (**K, L′′**) No clustering of Ifitm3-positive cells in the E7.5 *Dullard^−/−^* embryo. (**K, L**; whole embryo bright-field view; **K′, L′**; immunostaining, **K′′**, **L′′**; high magnification views of Iftim3-positive (red) cells in the boxed areas of **K′** and **L′**; DAPI nuclear staining (blue). (**M–N**) Marked reduction in the numbers of *Prdm1*-positive cells in the E7.25 streak-stage *Dullard^−/−^* embryo (**M′**, **N′**; high magnification views of the boxed areas in **M, N**, respectively). (**O–P′′**) Down-regulation of *Ifitm1* expression in the posterior mesoderm of the E7.5 *Dullard* ^−/−^ embryo [**O, P**, whole embryo; **O′, P′**, merged bright-field and immunofluorescence images (boxed area); **O′′, P′′**, immunofluorescence image only]. (**A, A′, C, C′, E, E′, G, I, K–K′′, M, M′, O–O′′**) *Dullard^ +/−^* embryos; (**B, B′, D, D′, F, F′, H, J, L–L′′, N, N′, P–P′′**) *Dullard^−/−^* embryos. (**E, F**) Dashed lines indicate the plane of sectioning. (**E′, F′**) Dashed lines mark the basal surface of the endoderm layer. Scale bars = 100 µm (**A–F′, K–L′, M–P′**′), 50 µm (**I, J**) and 25 µm (**K′′, L′**′).

Microarray analysis revealed a reduction in the expression of PGC-specific marker, *Dppa3/Pgc7/Stella*, in the *Dullard^−/−^* embryo (2.3-fold, Table S1). Q-PCR analysis also showed reduced *Dppa3* expression, while *Pou5f1* expression was unchanged (Fig. S4). In E7.75 (Fig. 2A, A′) and E8.5 (Fig. 2C, C′) *Dullard^+/−^* embryos, AP-positive PGCs were localized to a crescent-shaped domain in the endoderm at the margin of the prospective posterior intestinal portal. No AP-positive cells were found in the posterior endoderm of E7.75 (Fig. 2B, B′) and E8.5 (Fig. 2D, D′) *Dullard^−/−^* embryos. However, at E7.5, many AP-positive cells were present in the mesoderm and endoderm of the *Dullard^+/−^* embryo (Fig. 2E, E′), but only a few AP-positive cells were found in the mesoderm of *Dullard^−/−^* embryos (Fig. 2F, F′). Some AP-positive *Dullard^−/−^* cells displayed the characteristic staining pattern of *Dullard^+/−^* PGCs (Fig. 2G, H) and expressed Dppa3 (Fig. 2I, J). Thus, loss of Dullard function is associated with a severely reduced PGC population (Table 1).

**Table 1:** Genetic interactions between *Dullard, Bmp4* and *Wnt3* as assessed by the numbers of primordial germ cells (PGCs) formed in embryonic day (E)7.75 embryos.

Genotype	PGCs		No. ofembryo
	Markers	No.(mean±SEM)	
*Dullard+/+*	Dppa3	18.8±1.9^a^	11
*Dullard+/−*	Dppa3 AP	18.8±1.2 32.7±3.2^d^	13 10
*Dullard−/−*	Dppa3 AP	1.6±0.5^a,^ e 7.5±1.5^d,^ e	11 10
*Bmp4+/−*	Dppa3	10.2±1.2^b^	13
*Dullard+/−;Bmp4+/−*	Dppa3	11.9±1.1	16
*Dullard−/−;Bmp4+/−*	Dppa3	1.1±0.5^b^	9
*Wnt3+/−*	AP	27.5±0.2^c^	11
*Wnt3−/−*	Dppa3, AP,*Ifitm3*	0	5
*Dullard+/−;Wnt3+/−*	AP	15. 7±0.1^c^	20
*Dullard+/−; Wnt3−/−*	AP	0	2

a–d Pairs of results differ significantly; *P*<0.001, Student’s *t*-test.

e Cells in the mesoderm. AP, alkaline phosphatase. SEM, standard error of the mean.

To assess whether loss of Dullard affects the formation of PGC precursors, we examined the expression of *Prdm1* and Ifitm3 [1], [38]–[41]. In the E7.25 *Dullard^+/−^* embryo, many Ifitm3-positive precursor cells were clustered near the posterior end of the primitive streak (Fig. 2K–K′′; [28]). However, in the *Dullard^−/−^* embryo, fewer Ifitm3-positive cells were found (Fig. 2L–L′′). Similarly, *Prdm1*-positive precursor cells that clustered in the posterior epiblast of the E7.25 *Dullard* ^+/−^ embryo (Fig. 2M, M′; [1], [41]), were absent in the *Dullard^−/−^* embryo (Fig. 2N, N′). Thus, loss of Dullard impairs the formation of PGC precursors.

*Dullard^−/−^* AP-positive cells were unable to transit from the mesoderm to the endoderm as indicated by only one *Dullard^−/−^*embryo showing three AP-positive cells in the endoderm. In contrast, many AP-positive cells were localized in the endoderm of *Dullard^+/−^* embryos (Fig. 2E′). *Ifitm1,* which was expressed in PGC precursors and the posterior mesoderm in wild-type embryos (Fig. 2O–O′′, [39]), was not expressed in *Dullard^−/−^* embryos (Fig. 2P–P′′). Our previous study [19], [42] has shown that proper expression of *Ifitm1* is essential for navigating PGCs from the mesoderm to endoderm. Disruption of *Ifitm1* expression following the loss of Dullard may therefore have impeded the translocation of PGCs.

### Dullard does not Affect BMP4 Activity in PGC Formation

**Figure 3:**
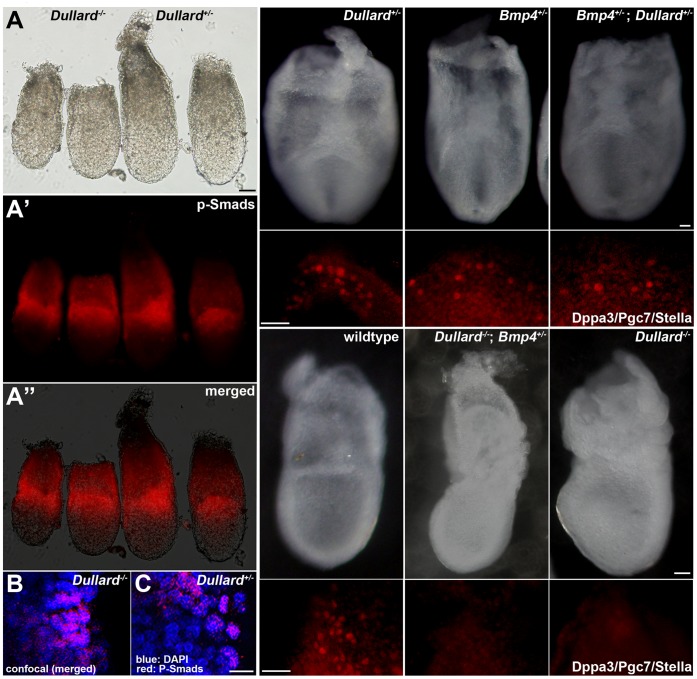
Dullard does not affect BMP4 signalling. (**A–A′′**) The expression pattern of p-Smads in the proximal epiblast and adjacent extraembryonic ectoderm of the E6.75 *Dullard* ^−/−^ embryo was similar to that in *Dullard* ^+/−^ embryos (**A**, whole embryo bright-field view; **A′**, immunofluorescence image; **A′′**, merged image; left pair, *Dullard* ^−/−^ embryos; right pair, *Dullard* ^+/−^ embryos). (**B, C**) Confocal images of immunostaining of p-Smads in *Dullard* ^−/−^ and *Dullard* ^+/−^ embryos, respectively. (**D–I′**) Formation of Dpp3/Pgc7/Stella-positive PGCs was unaffected by the combined loss-of-function of *Dullard* and *Bmp4*. (**D–F′**) E7.75 compound heterozygous *Dullard; Bmp4* embryos contained similar numbers of PGCs as those in *Dullard^+/−^* and *Bmp4^+/−^* embryos. (**G–I′**) Loss of *Bmp4* activity in addition to complete loss of *Dullard* function had no effect on the PGC phenotype of E7.75 *Dullard* ^−/−^ embryos. (**D–I)** Whole embryo bright-field image; (**D–G**, posterior view; **H, I,** lateral views). (**D′–I′**) Dppa3/Pgc7/Stella immunostaining of the embryo in panels **D–I**. Scale bars = 100 µm (**A–A′**′), 20 µm (**B, C**) and 50 µm (**D–I′**).

Considering the putative role of Dullard in modulation of BMP signalling activity, we examined the effect of loss of Dullard on BMP4 signalling activity. In the *Dullard^−/−^* embryo, *Bmp4* was expressed at the appropriate level (Fig. S2A) in extraembryonic tissue (Fig. S3R–T), and the expression of BMP signal transducers, *Smad1*, *Smad4* and *Smad5*, was unchanged (Table S1). p-Smads (the active form of Smad1, 5 and 8) were expressed appropriately in the proximal epiblast of E6.75 *Dullard^−/−^* and *Dullard^+/−^* embryos (Fig. 3A–C). Consistent with the normal expression of *Bmp4* and p-Smads, Q-PCR and in situ hybridization analyses showed appropriate expression of BMP downstream target genes, *Id1* and *Msx2* in E7.5 and E8.25 *Dullard^−/−^* embryos (Fig. S3U–W, S4, and data not shown). In the E7.5 *Bmp4* ^−/−^ embryo, *Dullard* expression was unchanged (Fig. S2). Therefore, loss of Dullard does not appear to affect BMP activity in embryos at gastrulation and early organogenesis.

To examine whether Dullard function intersects with BMP signalling in PGC formation specifically, we examined the effect of altering the Dullard gene dosage in *Bmp4^+/−^* embryos. In E7.75 *Bmp4* ^+/−^ embryos, fewer Dppa3-positive PGCs (Fig. 3E, E′; [9]) were found than those in *Dullard* ^+/−^ (Fig. 3D, D′; Table 1) and wild-type (Fig. 3G, G′, Table 1) embryos. Loss of one copy of the *Dullard* gene in *Bmp4* ^+/−^ embryos (i.e. *Dullard* ^+/−^; *Bmp4* ^+/−^ compound mutant) had no effect on the number of Dppa3-positive PGCs (Fig. 3F, F′), which was similar to that in *Bmp4* ^+/−^ embryos (Fig. 3E, E′; Table 1). Furthermore, the number of PGCs in E7.75 *Dullard* ^−/−^; *Bmp4* ^+/−^ embryos (Fig. 3H, H′) was similar to that in *Dullard^−/−^* embryos (Fig. 3I, I′; Table 1). These results suggest that, if Dullard is a negative regulator of BMP receptor activity, complete loss of its function does not compensate for the reduced BMP activity in *Bmp4* ^+/−^ embryos.

### Loss of Dullard Affects WNT Signalling Activity

**Figure 4:**
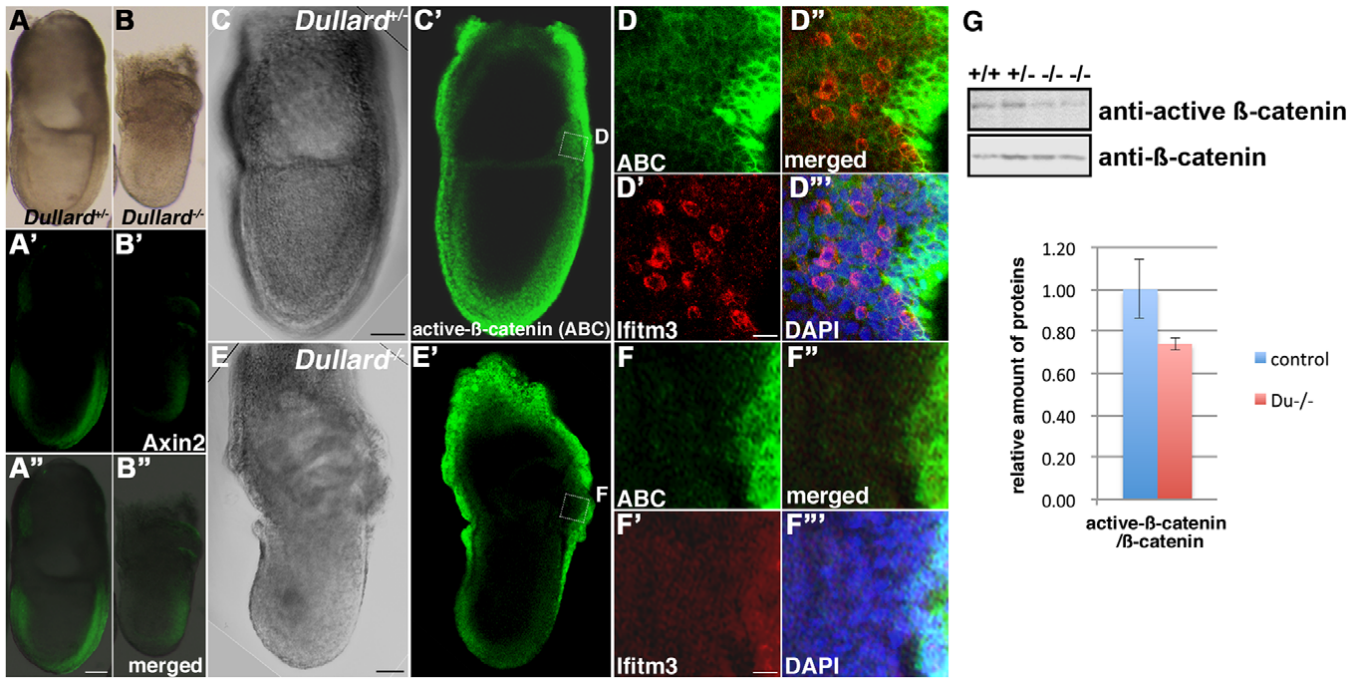
Down-regulation of WNT/β-catenin signalling activity in *Dullard* ^−/−^ embryos. (**A–B′′**) Curtailed expression of *Axin2* in the (**B–B′′**) E7.5 *Dullard* ^−/−^ embryo compared with that in the (**A–A′′**) *Dullard* ^+/−^ embryo. (**A, B**, bright-field images; **A′, B′**, Axin2 immunostaining; **A′′, B′′**, merged images). (**C–F′′′**) Expression of activated β-catenin (ABC) visualized by immunostaining and confocal microscopy of (**C–D′′′**) E7.5 no bud-stage *Dullard* ^+/−^ embryos and (**E–F′′′**) E7.5 *Dullard* ^−/−^ embryos. ABC expression in the posterior germ layer of the embryo, where Ifitm3-positive PGCs were localized, was weaker in the *Dullard^−/−^* embryo that also lacked Ifitm3-postive PGCs. (**C, E**) Bright-field image; (**C′, E′**) ABC immunostaining; (**D–D′′′, F–F′′′**) magnified views of the boxed areas in panels **C′** and **E′**, respectively; (**D′′, F′′**) merged ABC and Iftm3 images; (**D′′′, F′′′**) merged images with DAPI nuclear staining; ABC immunostaining (green); Ifitm3 immunostaining (red), Scale bar = 100 µm (A–C′, E–E′) and 20 µm (**D–D′′′, F–F′′′**). (**G**) Western blot analysis of E7.75 wild-type (+/+) and *Dullard^ +/−^* (+/−) embryos showing reduced amounts of the active form of β-catenin in *Dullard^−/−^* (−/−) embryos, but total β-catenin content was unchanged.

Canonical WNT signalling activity, acting in concert with BMP, has been shown to enhance the generation of the germ cell lineage from epiblast cells and ES cells in vitro [13], [43]. Further analysis of the expression profiling data of the E7.5 *Dullard^−/−^* embryo revealed a potential role of Dullard in modulation of WNT signalling activity (Table S1). Q-PCR analyses confirmed significant down-regulation of several WNT downstream genes and WNT targets including *Brachyury*, a WNT3 downstream target gene [44]; *Ifitm1*, a WNT/β-catenin-dependent downstream gene [45], [46], and *Nkx1-2*, which responds to WNT/β-catenin signalling and activates *Brachyury* expression through the suppression of *Tcf3* (in P19 mouse embryonal carcinoma cells; [47]) in the E7.5 *Dullard^−/−^* embryo (Fig. S4). The expression of other WNT/β-catenin-dependent targets, *Lef1* and *Axin2*/*conductin*, was significantly reduced as indicated by Q-PCR (Fig. S4). Reduced expression of WNT-response genes was also found by in situ hybridization and whole mount immunostaining (*Brachyury*, S3E–F′; Ifitm1, Fig. 2O–P′′; Axin2, Fig. 4A–B′′). Expression of active β-catenin was reduced in the posterior epiblast of the E7.5 *Dullard^−/−^* embryo, where Ifitm3-positive PGCs were found (Fig. 4C–F′′′). As shown by western blotting, *Dullard^−/−^* embryos expressed a similar amount of β-catenin as that in E7.75 control embryos, but the amount of active β-catenin was reduced in mutants (n = 2, Fig. 4G). Taken together, these findings indicate that loss of Dullard is associated with a reduction in WNT/β-catenin signalling activity.

### *Wnt3* Interacts with *Dullard* in PGC Formation

**Figure 5:**
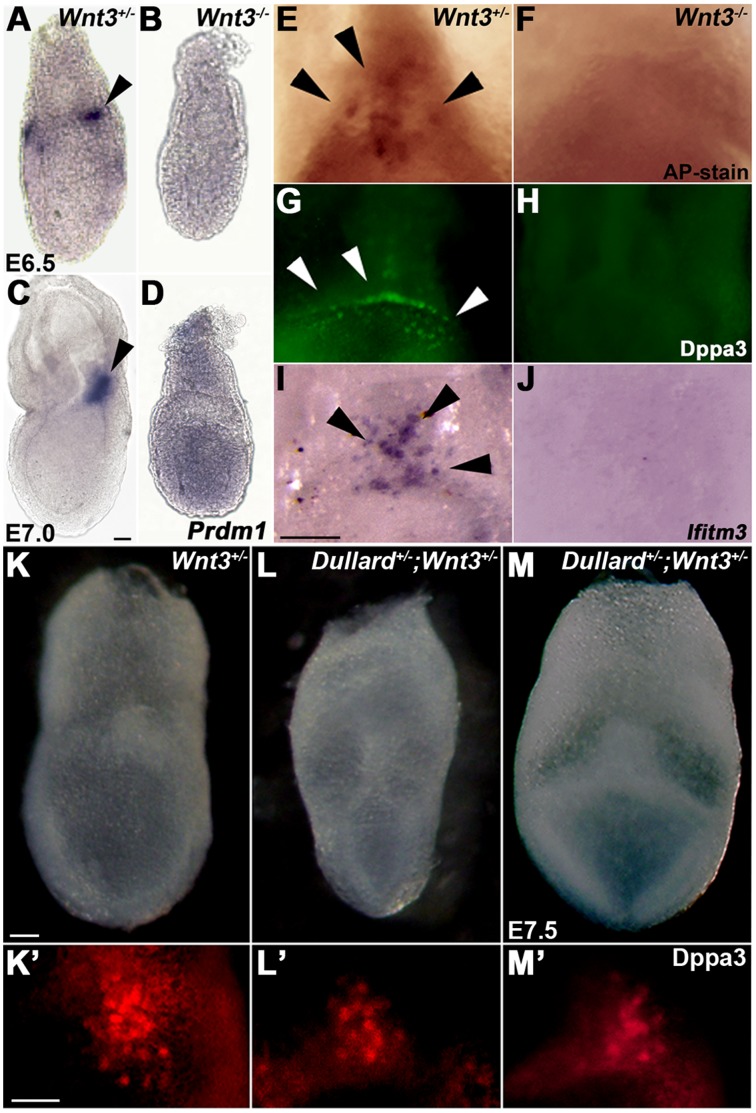
Wnt3 signalling is required for PGC formation. (**A–D**) Diffuse pattern of *Prdm1* expression in E6.5 (**B**) and E7.0 (**D**) *Wnt3^−/−^* embryos and absence of the cluster of *Prdm1*-positive PGC progenitors compared with that in (**A, C**) *Wnt3* ^+/−^ counterparts. (**E–J**) Absence of PGCs that were marked by AP (**E, F**), Dppa3 (**G, H**) and *Ifitm3* (**I, J**) in *Wnt3^−/−^* embryos compared with that in *Wnt3^+/−^* embryos (arrowhead: PGCs). (**E, F, I, J**) E7.75 and (**G, H**) E8.0 embryos. (**K–M′**) Reduced number of *Dppa3*-expressing PGCs in *Dullard; Wnt3* compound mutant embryos. (**K–M**) Bright-field images of E7.75 (**K**) *Wnt3* ^+/−^ and (**L, M**) *Dullard* ^+/−^; *Wnt3* ^+/−^ embryos (posterior view). (**K′–M′**) Dppa3/Pgc7/Stella immunostaining of PGCs in the posterior region of embryos shown in **K–M**. Scale bars = 100 µm.

*Wnt3* is known to play an essential role in the initiation of gastrulation [26] and PGC formation [13] In E6.5 and E7.0 wild-type embryos, *Prdm1-*expressing PGC precursors were localized as a tight cluster in the posterior epiblast (Fig. 5A, C; [1], [41]. Conversely, in E6.5 and E7.0 *Wnt3^−/−^* embryos, *Prdm1* was expressed widely in the epiblast and not in any cell cluster (Fig. 5B, D; [13]). There were no Dppa3-, *Ifitm3-* or AP-positive PGCs in E7.75–E8.0 *Wnt3^−/−^* embryos (Fig. 5E–J; [13]). In the *Wnt3^−/−^* mutant embryo, *Dullard* expression was unchanged, but *Bmp4* expression was markedly reduced (Fig. S2A).

**Figure 6:**
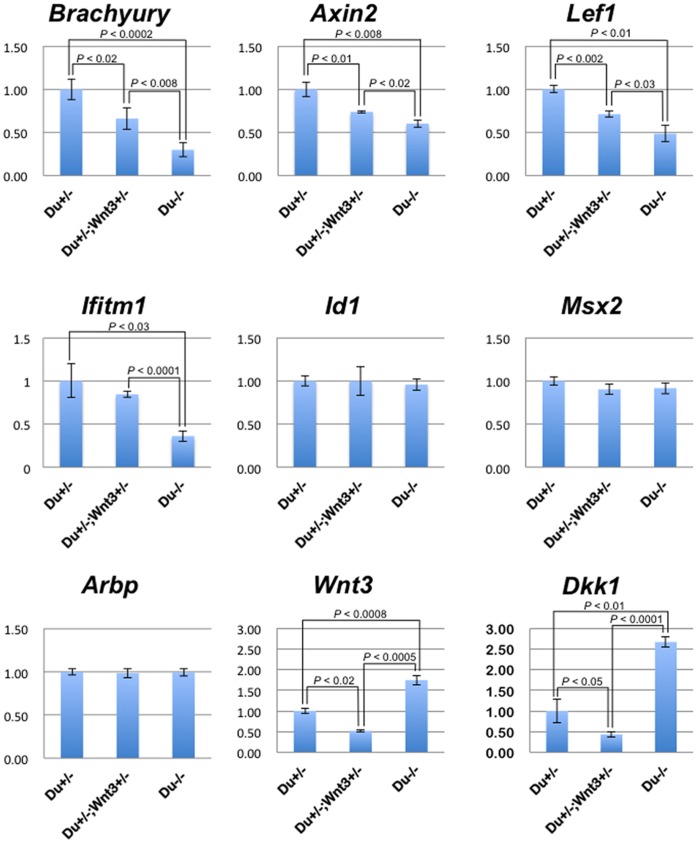
*Dullard* ^+/−^; *Wnt3* ^+/−^ embryos show intermediate levels of WNT/β-catenin- dependent downstream gene expression. In Q-PCR analyses, E7.5 *Dullard* ^−/−^ embryos (Du^−/−^) showed significant down-regulation of WNT/β-catenin-dependent downstream genes: *Brachyury* (*P*<0.0002, Student’s *t*-test), *Axin2* (*P*<0.008), *Lef1* (*P*<0.01) and *Ifitm1* (*P*<0.03), compared with that in *Dullard* ^+/−^ embryos (Du^+/−^). *Dullard* ^+/−^; *Wnt3* ^+/−^ embryos (Du^+/−^; Wnt3^+/−^) showed significant down-regulation of WNT/β-catenin- dependent downstream genes*: Brachyury* (*P*<0.02), *Axin2* (*P*<0.01) and *Lef1* (*P*<0.002) compared with that in Du^+/−^, and also showed significant up-regulation of *Brachyury* (*P*<0.008), *Axin2* (*P*<0.02), *Lef1* (*P*<0.03) and *Ifitm1* (*P*<0.001) compared with that in Du^−/−^. Expression of BMP downstream target genes (*Id1* and *Msx2*) was unchanged. Du^−/−^ showed significant up-regulation of *Wnt3* (*P*<0.0008) and *Dkk1* (*P*<0.01) compared with that in Du^+/−^. Du^+/−^; Wnt3^+/−^ showed significant down-regulation of *Wnt3* and *Dkk1* compared with that in Du^+/−^ [*Wnt3* (*P*<0.02) and *Dkk1* (*P*<0.05)] and Du^−/−^ [*Wnt3* (*P*<0.0005) and *Dkk1* (*P*<0.001)], respectively. mRNA expression levels were normalized to those of *Gapdh* (internal control), and the levels in *Dullard* ^+/−^ embryos (≈ control) were set to 1. *Arbp*, internal control.

To test whether Dullard mutation influences the effect of loss of *Wnt3* on PGC formation, we studied the effect of genetic interactions in *Wnt3; Dullard* compound mutant embryos using WNT-response gene expression and PGC formation as experimental indicators. Reduced Dullard activity had a synergistic effect on PGC formation on a *Wnt3^+/−^* background. *Dullard* ^+/−^; *Wnt3* ^+/−^ embryos contained about half of the number of Dppa3- and AP-positive PGCs as that in wild-type embryos. In contrast to the *Dullard^−/−^* embryo, the compound heterozygous mutant embryo did not show any morphological defects (Fig. 5K–M′, Table 1). These findings suggest that the effect on PGC formation may be unrelated to the developmental abnormalities associated with loss of Dullard, but may be the consequence of altered WNT activity. In E7.5 *Dullard* ^+/−^ *; Wnt3* ^+/−^ embryos, *Dullard* and *Wnt3* were expressed at 70% and 35% of the wild-type level, respectively (Fig. S2A). E7.5 *Wnt3* ^+/−^ and *Dullard^+/−^* embryos showed similar expression levels of WNT/β-catenin downstream genes, *Brachyury, Ifitm1, Axin2* and *Lef1* (Fig. S2B), whereas *Dullard* ^+/−^ *; Wnt3* ^+/−^ embryos displayed a significantly lower level of downstream gene activity than that in *Dullard^+/−^* embryos (except for *Ifitm1*), but was higher than that in *Dullard^−/−^* embryos (Fig. 6).

### *Dullard* Fine-tunes WNT Activity that is Critical for PGC Formation

Because WNT response gene expression was reduced in the *Dullard^−/−^* embryo, we next examined the expression of WNT signal pathway genes to elucidate the mechanism of WNT activity modulation by Dullard. Results of the microarray analysis of *Dullard^−/−^* embryos showed that the expression of many WNT factors (*Wnt1, Wnt2b, Wnt4, Wnt5A, Wnt6, Wnt7a, Wnt7bB, Wnt8a, Wnt11*, and *Wnt16*) did not change significantly (<1.2-fold changes, Table S1). However, some WNT factors were down-regulated (*Wnt8b*: 1.7-fold decrease; *Wnt3a*: 2.0-fold decrease; *Wnt10a*: 2.0-fold decrease), whereas others were up-regulated (*Wnt3*∶1.6-fold increase; *Wnt10b*: 1.7-fold increase). Among the WNT pathway components that responded to the loss of Dullard, *Wnt3* might be one of the critical factors, because ablation of one *Wnt3* allele (*Dullard* ^+/−^; *Wnt3* ^+/−^ compound mutants) resulted in a further reduction of WNT-response gene expression (Fig. 6) and enhanced the loss of PGCs (Fig. 5K–M′, Table 1). Among the antagonists, *Dickkopf-1* (*Dkk1*; 1.3-fold increase), *secreted frizzled-related protein-1* (*Sfrp1*; 1.5-fold increase) and *Sfrp5* (2.0-fold increase) were up-regulated, while *Sfrp2, Sfrp4* and *Frzb*/*Sfrp3* expression was unchanged. The expression of WNT receptors, *low density lipoprotein receptor-related protein-5* (*Lrp5*) and *Lrp6*, and intracellular signal transducer *Dishevelled* family genes (*Dvl1, 2 and 3*) was unchanged (Table S1).

The enhanced *Wnt3* expression in *Dullard^−/−^* embryos was verified by Q-PCR (Fig. 6), in situ hybridization (Fig. S5C, D), and immunostaining using an anti-WNT3/3a antibody (Fig. S5A–B′). *Wnt3* activity has been shown to be antagonized mainly by *Dkk1* during early post-implantation development [48]. *Wnt3* signalling is required to activate *Dkk1*, a direct WNT3 target [49], and in return, WNT3 is antagonised by *Dkk1* to deliver the proper level of signalling activity required for the formation of the embryonic head [48]. Q-PCR results showed that the 1.8-fold increase of *Wnt3* expression in the *Dullard^−/−^* embryo was accompanied by a more robust increase in *Dkk1* expression (2.7-fold) (Fig. 6) as well as enhanced expression of two other WNT antagonists, *Sfrp1* and *5* (Fig. S4). This high level of antagonistic activity would have effectively nullified the elevated *Wnt3* signalling activity in the mutant embryo, resulting in an overall reduction of the WNT downstream activity.

In contrast to the *Dullard* ^−/−^ embryo that expressed *Wnt3* at 1.8-fold and *Dkk1* at 2.7-fold of the respective levels in *Dullard* ^+*/−*^ embryo, the *Dullard* ^+/−^; *Wnt3* ^+/−^ embryo expressed *Wnt3* and *Dkk1* (*Wnt3*∶0.5-fold; *Dkk1*∶0.4-fold) at about half of the *Dullard* ^+/−^ level (Fig. 6). The more balanced activity of the agonist and the antagonist may achieve a level of WNT signalling that was permissible for PGC formation (Fig. 5K–M′, Table 1). Supporting this concept, in E7.5 *Dullard^+/−^; Wnt3^+/−^* embryos, the expression levels of WNT/β-catenin downstream genes were between those in *Dullard* ^+/−^ and *Dullard^−/−^* embryos (Fig. 6). E7.75 *Dullard^+/−^; Wnt3^+/−^* embryos contained about half of the normal number of AP-positive PGCs (Table 1). Collectively, these findings show that Dullard may fine-tune the overall level of WNT activity through concerted control of the activities of *Wnt3* and *Dkk1*.

### Dullard Interacts with Dishevelled

**Figure 7:**
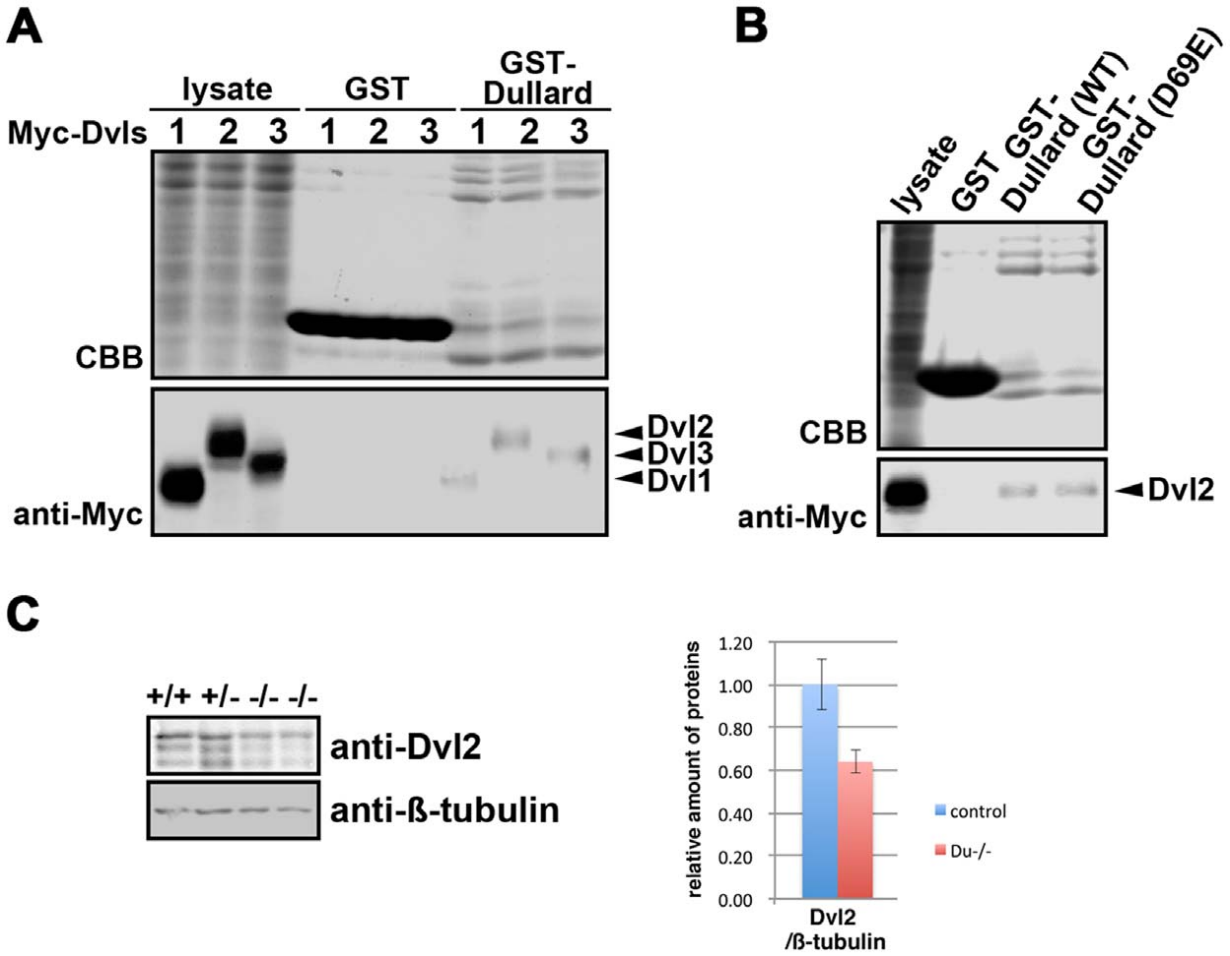
Effect of loss of Dullard function on Dishevelled (Dvl) proteins. (**A**) Glutathione S-transferase (GST) pull-down assays revealed that Dullard formed a protein complex with Dvls in HEK293 cells. The association of Myc-tagged Dvls with the GST-Dullard recombinant protein (arrowheads) was detected by an anti-Myc antibody. CBB, Coomassie blue bands. (**B**) GST-Dullard with a D69E mutation in the catalytic domain, which abolishes its phosphatase activity, showed a binding activity with Myc-tagged Dvl2 similar to that of the wild-type (WT) Dullard protein. (**C**) Western blot analysis showing less Dvl2 protein in *Dullard^−/−^* embryos. β-tubulin, loading control.

Results of the genetic study suggested that Dullard may act as a positive regulator of WNT signalling activity. To test whether Dullard acts at another juncture in the WNT cascade, a GST-pull down assay was performed in HEK293 cells to identify Dullard-interacting proteins that are involved in signal transduction. A GST-Dullard recombinant protein (lacking the first 30 amino acids; [16]) interacted with Myc-tagged Dvl1, Dvl2 and Dvl3 (Fig. 7A), which are members of the intracellular WNT signal transducer family. A GST-Dullard protein harbouring the D69E mutation in the catalytic domain, which abolishes its phosphatase activity [15], [16], also displayed a binding activity with Myc-tagged Dvl2 similar to that of the control counterpart (Fig. 7B). Phosphatase activity of Dullard is therefore not required for the interaction with Dvl2.

**Figure 8:**
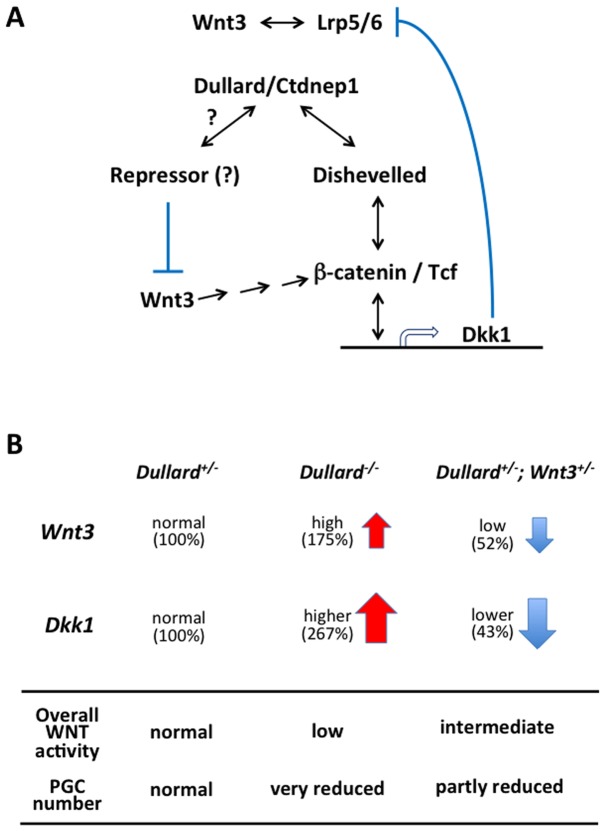
Intersection of Dullard function with WNT signalling activity . (**A**) Dullard interacts with Dishevelled to influence the transduction of signals that regulate the expression of downstream genes including *Dkk1* and *Axin2*. Dkk1 antagonizes WNT3 signalling activity by interacting with the Lrp5/6 co-receptor upstream of the signal transducing pathway involving Dishevelled. Dullard may interact with a repressor molecule that negatively regulates the expression of *Wnt3*, which signals through the β-catenin/Tcf dependent cascade (via a multi-step process: arrows) to activate *Dkk1* expression. (**B**) Profiles of *Wnt3* (agonist) and *Dkk1* (antagonist) expression as well as overall signalling activity for PGC formation. The fine-tuning of WNT/β-catenin signalling activity by Dullard is crucial for PGC formation. Loss of Dullard function produced high *Wnt3* activity (175% of the heterozygous level) that may be effectively nullified by higher *Dkk1* activity (267%). This occurrence would lead to the overall reduced (low) WNT signalling activity that was accompanied by loss of PGCs. In the *Dullard* ^+/−^; *Wnt3* ^+/−^ embryo, moderate *Wnt3* activity (52% of the heterozygous level: low) in the presence of lower (43%) *Dkk1* activity may establish an overall intermediate level of WNT activity that permits the formation of a smaller number of PGCs (partly restored PGC numbers).

Less Dvl2 protein was present in E7.75 *Dullard^−/−^* embryos compared with *Dullard^+/−^* embryos (n = 2, Fig. 7C), while expression of *Dvl1-3* mRNA was unchanged (Table S1). Therefore, loss of Dullard, which may act as a Dvl-interacting protein, may reduce the availability of Dvl2 protein and negatively affect WNT signal transduction activity (Fig. 8A) in conjunction with its potential role in balancing agonist and antagonist activities (Fig. 8B).

## Discussion

In this study, we have shown that loss of Dullard function in the mouse embryo phenocopies the PGC deficiency associated with the loss of BMP4 and WNT3 signalling activities. *Dullard,* which encodes a protein with phosphatase activity, is reputed to negatively regulate BMP signalling by dephosphorylation that inhibits the function of BMP receptors. Therefore, loss of Dullard is anticipated to result in enhanced BMP receptor activity, which may counteract the effect of absent or reduced BMP signalling. However, the results of our genetic study showed that *Dullard* has neither a synergistic nor an opposing effect on BMP4 activity in PGC formation. However, a possible role of Dullard in BMP signalling for PGC formation cannot be completely ruled out, and Dullard functions may intersect with BMP signalling in other developmental processes.

Similar to the effect of loss of *Wnt3* on PGC formation, as shown in our study and a previous study [13], PGCs are absent in *Dullard*-null mutant embryos, even though BMP signalling activity is unabated and the mutant cells remain responsive to BMP4 induction. It has been proposed that WNT3 may influence the competency of epiblast cells at E5.5 to respond to BMP4 induction of *Prdm1*-positive germ cell progenitors [13]. Although WNT alone is insufficient to induce the differentiation of epiblast cells into PGCs, induction of PGCs by BMP can be enhanced by WNT3A [13]. A combination of WNT and BMP signalling activity also induces the generation of PGCs from ES cells and induced pluripotent stem cells [43]. These findings suggest that WNT signalling acts to render epiblast cells competent to respond to BMP induction. The results of this study show that Dullard may function as an agonist of WNT activity. Loss of Dullard activity down-regulates WNT-response gene expression and the level of active β-catenin, which may disrupt the competency of epiblast cells to generate PGCs.

The induction of PGCs by WNT and BMP appears to require an intermediate step of differentiation into epiblast-like cells [43]. In vivo, the generation of *Prdm1*-positive cells from epiblast cells may continue through E6.5 to E7.25 [50]. In addition, distal epiblast cells in E6.5 embryos at the early primitive streak stage, which would not have experienced strong WNT or BMP signals, can give rise to the AP-positive PGCs when transplanted heterotopically into the proximal region [51]. This observation suggests that epiblast cells are still responsive to WNT and BMP signalling well after the initial phase of induction of PGCs. Therefore, WNT signalling activity may have a continuous influence on PGC formation beyond E5.5 to after the onset of gastrulation.

The action of Dullard on components of the WNT signalling cascade may be related to regulation of signalling activity through intricate positive and negative feedback regulatory loops [52]–[56]. Loss of Dullard resulted in reduced expression of some WNT ligands (*Wnt3A*, *Wnt8b*, and *Wnt10a*) and antagonists (*Dkk1, Sfrp1*, and *Sfrp5*), but paradoxically led to up-regulation of *Wnt3*, the functionally dominant ligand that influences early post-implantation embryonic development [26], [48]. The gain in WNT3 activity in the *Dullard^−/−^* embryo may be nullified by the more robust activity of the antagonist, Dkk1, which is a direct transcriptional target of WNT/β-catenin signals and negatively modulates signalling activity through its interaction with the receptor complex. The activation of this regulatory feedback loop may offset the effect of *Wnt3* up-regulation, thereby recapitulating a reduced overall WNT activity, such as that under a partial “*Wnt3*-null” (hypomorphic) condition. In this regard, reduction of the gene dosage of *Wnt3* in the *Dullard^+/−^* embryo may result in more balanced *Wnt3* and *Dkk1* activities, which still results in an overall decrease in signalling activity, as revealed by the decreased activation of WNT downstream genes (intermediate), but allows partial restoration of PGC formation (Fig. 8B). This proposed scenario highlights that the fine-tuning of WNT/β -catenin signalling activity by Dullard is crucial for PGC formation.

It is possible that Dullard may regulate WNT signal-related gene expression (Fig. 8A) in cooperation with other transcriptional partner(s). Other Dullard-related phosphatases that contain the DXDX(T/V) amino acid signature of the SCP/FCP family, such as the eyes absent homolog protein, can regulate gene expression through the formation of complexes with *sine oculis*-related homeobox protein homolog (Six) DNA binding proteins in developing organs including the eye, ear, muscle and kidney [57]. In yeast cells, nuclear envelope morphology protein 1 (Nem1) is required for protein complex formation with Sporulation-specific protein 7 (Spo7p) to dephosphorylate the target substrate, nuclear membrane-associated phosphatidic acid Smp2p [58]. Human *DULLARD* is able to rescue the aberrant nuclear envelope phenotype of *Nem1*-deficient yeast cells by dephosphorylation of Smp2p [16]. Recombinant human DULLARD is unable to dephosphorylate Lipin, a predicted mammalian ortholog of Smp2p, whereas DULLARD dephosphorylates mouse Lipin 1b only when it is over-expressed in BHK cells, but not in HeLa or HEK293A cells, suggesting that the mammalian counterpart of yeast Spo7p might be expressed in a cell type-specific manner [16]. In the *Dullard^−/−^* mouse embryo, the WNT signalling activity, as revealed by active β-catenin localization, was down-regulated most robustly in the posterior region of the embryo, where PGCs and their precursors are localized (Fig. 4C–F′′′). This result raises the possibility that the action of Dullard depends on its interaction with stage/site/cell-type specific partner protein(s) for targeting to the appropriate substrate for dephosphorylation.

The action of Dullard on other components of the WNT signalling cascade was revealed by the physical interaction between Dullard and Dvl proteins of the signal transduction cascade (Fig. 8A). The precise effect of the Dullard-Dvl interaction is presently unknown, but the amount of Dvl2 protein was reduced in *Dullard* ^−/−^ embryos. Dvl protein turnover is regulated by proteasomal and lysosomal degradation. Several Dvl-interacting proteins have been reported to facilitate Dvl poly-ubiquitination [59]. It is possible that binding to Dullard stabilizes the Dvl2 protein by inhibiting the binding of other Dvl-interacting protein(s) that facilitate ubiquitination and degradation. Therefore, the interaction with Dullard may ensure the availability of Dvl proteins for the transduction of WNT signals to effectors.

In conclusion, Dullard exhibits putative modulation of WNT signalling activity through regulation of WNT ligand/antagonist expression and Dvl2 protein availability for PGC formation. Although the results of our study are consistent with Dullard acting in a cell-autonomous manner, its effect on cell–cell interactions via the modulation of WNT signalling and its downstream activity remains to be elucidated.

## Supporting Information

Figure S1 **Strategy of gene targeting to generate modified** ***Dullard*** **alleles.** (**A–C**) A *Dullard-null* allele was generated in embryonic day (E)14.1 embryonic stem (ES) cells (129/ola) by replacing a 9.4 kb genomic region on chromosome 11 containing all 8 exons and introns with a *MC1-Neo* cassette (*Dullard^+/−^* ES cells). (**D–F**) TT2 ES cells (C57BL/6×CBA strains) containing a *Dullard-LacZ* allele (*Dullard^+/LacZ^*) were generated (http://www.cdb.riken.jp/arg/Methods.html; Murata et al., Gene Expr Patterns 5∶171-178, 2004) by replacing part of the 1^st^ exon and 2^nd^ to 4^th^ exons, which contain the DLDET catalytic domain of the phosphatase, before the initiation codon with a *lox71-LacZ-pA-frt-Pro-Neo-frt-loxP-pA* cassette (http://www.cdb.riken.jp/arg/cassette.html). Expression of *Dullard* was reported by *LacZ* expression. (**B, E**) The homologous recombination event was confirmed by Southern blotting with 5′ and 3′ external probes. (**B, C, F)** Chimeric mice, derived from two independent clones each of *Dullard^+/−^* and *Dullard^+/LacZ^* ES cells, were crossed with C57BL/6 mice to generate heterozygous mice. Genotypes of the offspring of germ-line chimeric embryos were determined by polymerase chain reaction (PCR) amplification of genomic DNA. Positions of primer sets for detection of the wild-type allele (DuS and DuAS) and mutant allele (Neo-b and Du2) in *Dullard^+/−^* offspring and the wild-type allele (DuL13 and RD-GR) and mutant allele (RD-F and LZ-R) in *Dullard^+/LacZ^* offspring are indicated by the arrowheads (see **A**, **D**). Primer sequences were: DuS: 3′-gttcttgggacaccgtctgt-5′, DuAS: 3′-agtcctgcctctttcaccaga-5′, Neo-b: 3′-gcgttggctacccgtgatat-5′, Du2∶3′-ttacaggtatgggggattgg-5′, DuL13∶3′-atgatgcggacgcagtgtctgc-5′, RD-GR: 3′-gaaccttgcttaaaggtgtcc-5′, RD-F: 3′-actccgtgctcatctctgcag-5′ and LZ-R: 3′-attcaggctgcgcaactgttgg-5′.
(TIF)

Figure S2 ***Dullard*** **expression is unaffected by loss of BMP4 or WNT3 functions.** (**A**) Q-PCR analyses of E7.5 *Dullard*-, *Bmp4*- and *Wnt3*-mutant embryos revealed that the expression levels of *Dullard* in *Wnt3^−/−^* and *Dullard^ +/−^*; *Wnt3^+/−^* embryos were similar to those in the *Dullard^ +/−^* embryo. The *Bmp4^−/−^* embryo expressed *Dullard* at a level similar to that in the wild-type embryo. *Wnt3* was significantly up-regulated in the *Dullard^−/−^* embryo (*P*<0.02, Student’s *t* test). *Bmp4^−/−^* embryos showed similar *Wnt3* expression levels as that in the *Dullard^ +/−^* embryo. The expression level of *Bmp4* was unchanged in *Dullard^ +/−^* and *Dullard^−/−^* embryos. mRNA expression levels were normalized to those of *Gapdh* (internal control), and the levels in wild-type (control) embryos were set to 1. *Arbp*, internal control. (**B**) *Wnt3* ^+/−^ and *Dullard^+/−^* embryos showed similar WNT/β-catenin dependent downstream activity. Expression of *Brachyury, Ifitm1, Axin2* and *Lef1* were not significantly different (*P*>0.1) between E7.5 *Wnt3* ^+/−^ (Wnt+/−) and *Dullard^+/−^* (Du+/−) embryos. *Wnt3* expression in Wnt+/− embryos was about 75% of that in Du+/− embryos (*P*>0.1). *Dkk1* expression in Wnt+/− embryos was reduced to about 68% of that in Du+/− embryos (*P*<0.07, n = 3 independent embryos). mRNA expression levels were normalized to those of *Gapdh* (internal control), and the levels in Du+/− embryos were set to 1. *Arbp*, internal control.
(TIF)

Figure S3 **Loss of Dullard function alters the expression domain of germ layer marker genes but does not affect embryonic patterning or the formation of mesoderm.** Compared with *Dullard^ +/−^* embryos, *Dullard^−/−^* embryos showed a similar expression pattern of (A, B) *Cer1* in the anterior visceral endoderm at E6.5. (C) *Lhx1* expression in the posterior and anterior axial mesoderm of the mid streak-stage embryo. (D) *Eomes* expression in the mesoderm (and also in the extraembryonic ectoderm, arrowhead) of the mid streak-stage embryo. (E, F) *Brachyury* expression in the primitive streak and nascent mesoderm (but no anterior extension of the expression domain) of the late streak-stage embryo. (E′, F′: transverse sections showing weaker expression in the primitive streak of the null mutant). (G, H) In the late bud-stage embryo, *Fgf8* was expressed strongly in the anterior primitive streak but weakly in the posterior segment (arrowhead). (I, J) *Flk1* was expressed in the embryonic mesoderm but absent in the extraembryonic mesoderm (arrowhead) of the E7.75 head fold-stage embryo. (K) *Id2* was expressed in the extraembryonic ectoderm (arrow) of the E7.5 *Dullard^−/−^* embryo, where *Eomes* was also expressed (see D, arrow). (L–W) *Dullard^−/−^* embryos failed to form the allantois and amnion, and showed accumulation of *Flk1*-, *Bmp4*- and *Id1*-expressing cells in their place. In situ hybridization on sections of *Dullard^−/−^* embryos for *Brachyury* (L–N), *Flk1* (O–Q), *Bmp4* (R–T) and *Id1* (U–W). (L, O, R, U) E8.25 *Dullard^−/−^* embryos; (M, P, S, V) E8.25 *Dullard^ +/−^* littermates and (N, Q, T, W) E7.5 *Dullard^ +/−^* embryos. Pro-Dis, proximal–distal axis; Ant-Pos, anterior–posterior axis. Scale bars = 100 µm.
(TIF)

Figure S4 **Expression of mesoderm, PGC-related and WNT antagonist genes in E7.5** ***Dullard^−/−^*** **embryos.** Q-PCR analyses of E7.5 embryos revealed that *Gsc* (*P*<0.02, Student’s *t*-test), *Sfrp1* (*P*<0.05) and *Sfrp5* (*P*<0.05) were up-regulated, whereas *Brachyury* (*P*<0.02), *Nkx2-1* (*P*<0.01), *Ifitm1* (*P*<0.02), *Lef1* (*P*<0.02), *Axin2* (*P*<0.002) and *Dppa3* (*P*<0.03) were down-regulated in *Dullard^−/−^* embryos (Du*−/−*), compared with that in wild-type embryos (+/+) (n = 3 independent embryos). *Id1* and *Msx2* (BMP downstream target genes), *Pou5f1* (stem cell marker gene) and *Arbp* (internal control) expression was unchanged. mRNA expression levels were normalized to those of *Gapdh* (internal control), and the levels in wild-type (control) were set to 1.
(TIF)

Figure S5 **Altered** ***Wnt3*** **/WNT3 expression in** ***Dullard*** **^–/–^ embryos.** Immunofluorescence analysis revealed (**A, A′**) the regionalized expression of WNT3 (and WNT3A) in the anterior two-thirds of the primitive streak of E7.5 *Dullard* ^+/–^ embryos. (**B, B′**) Expanded domain of WNT3 (and WNT3A) expression in the posterior germ layers of the E7.5 *Dullard* ^–/–^ embryo. (**A, B**) Bright-field images; (**A′, B′**) immunostaining with an anti-WNT3/3A antibody. (**C, D**) In situ hybridization analysis showing the broad domain of *Wnt3* expression in the posterior germ layers of (**C**) E6.5 and (**D**) E7.5 *Dullard* ^–/–^ embryos. Scale bars = 100 μm.
(TIF)

Table S1 **Microarray analysis of the expression of genes related to BMP signalling, WNT signalling and PGC formation in E7.5** ***Dullard^+/−^*** **embryos versus** ***Dullard^+/−^*** **embryos.**
(DOCX)
