# Supplementary figures and images for: Dullard/Ctdnep1 Modulates WNT Signalling Activity for the Formation of Primordial Germ Cells in the Mouse Embryo
Source: PLoS One. 2013 Mar 4;8(3):e57428. doi: 10.1371/journal.pone.0057428 (PMC3587611; doi:10.1371/journal.pone.0057428)

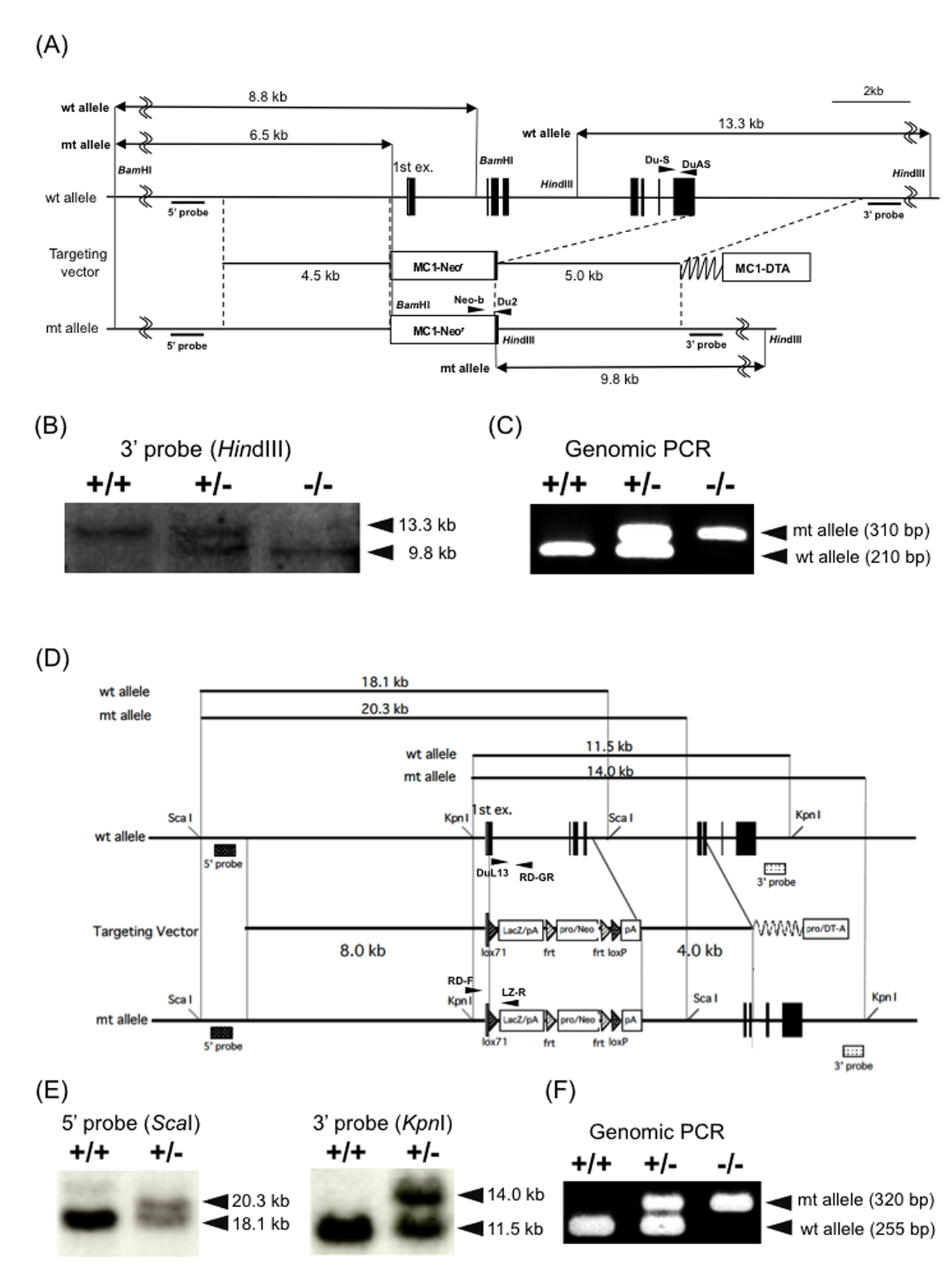

Supplement: Figure S1 — Strategy of gene targeting to generate modified Dullard alleles. (A–C) A Dullard-null allele was generated in embryonic day (E)14.1 embryonic stem (ES) cells (129/ola) by replacing a 9.4 kb genomic region on chromosome 11 containing all 8 exons and introns with a MC1-Neo cassette (Dullard+/− ES cells). (D–F) TT2 ES cells (C57BL/6×CBA strains) containing a Dullard-LacZ allele (Dullard+/LacZ) were generated (http://www.cdb.riken.jp/arg/Methods.html; Murata et al., Gene Expr Patterns 5∶171-178, 2004) by replacing part of the 1st exon and 2nd to 4th exons, which contain the DLDET catalytic domain of the phosphatase, before the initiation codon with a lox71-LacZ-pA-frt-Pro-Neo-frt-loxP-pA cassette (http://www.cdb.riken.jp/arg/cassette.html). Expression of Dullard was reported by LacZ expression. (B, E) The homologous recombination event was confirmed by Southern blotting with 5′ and 3′ external probes. (B, C, F) Chimeric mice, derived from two independent clones each of Dullard+/− and Dullard+/LacZ ES cells, were crossed with C57BL/6 mice to generate heterozygous mice. Genotypes of the offspring of germ-line chimeric embryos were determined by polymerase chain reaction (PCR) amplification of genomic DNA. Positions of primer sets for detection of the wild-type allele (DuS and DuAS) and mutant allele (Neo-b and Du2) in Dullard+/− offspring and the wild-type allele (DuL13 and RD-GR) and mutant allele (RD-F and LZ-R) in Dullard+/LacZ offspring are indicated by the arrowheads (see A, D). Primer sequences were: DuS: 3′-gttcttgggacaccgtctgt-5′, DuAS: 3′-agtcctgcctctttcaccaga-5′, Neo-b: 3′-gcgttggctacccgtgatat-5′, Du2∶3′-ttacaggtatgggggattgg-5′, DuL13∶3′-atgatgcggacgcagtgtctgc-5′, RD-GR: 3′-gaaccttgcttaaaggtgtcc-5′, RD-F: 3′-actccgtgctcatctctgcag-5′ and LZ-R: 3′-attcaggctgcgcaactgttgg-5′. (TIF) [file pone.0057428.s001.tif]

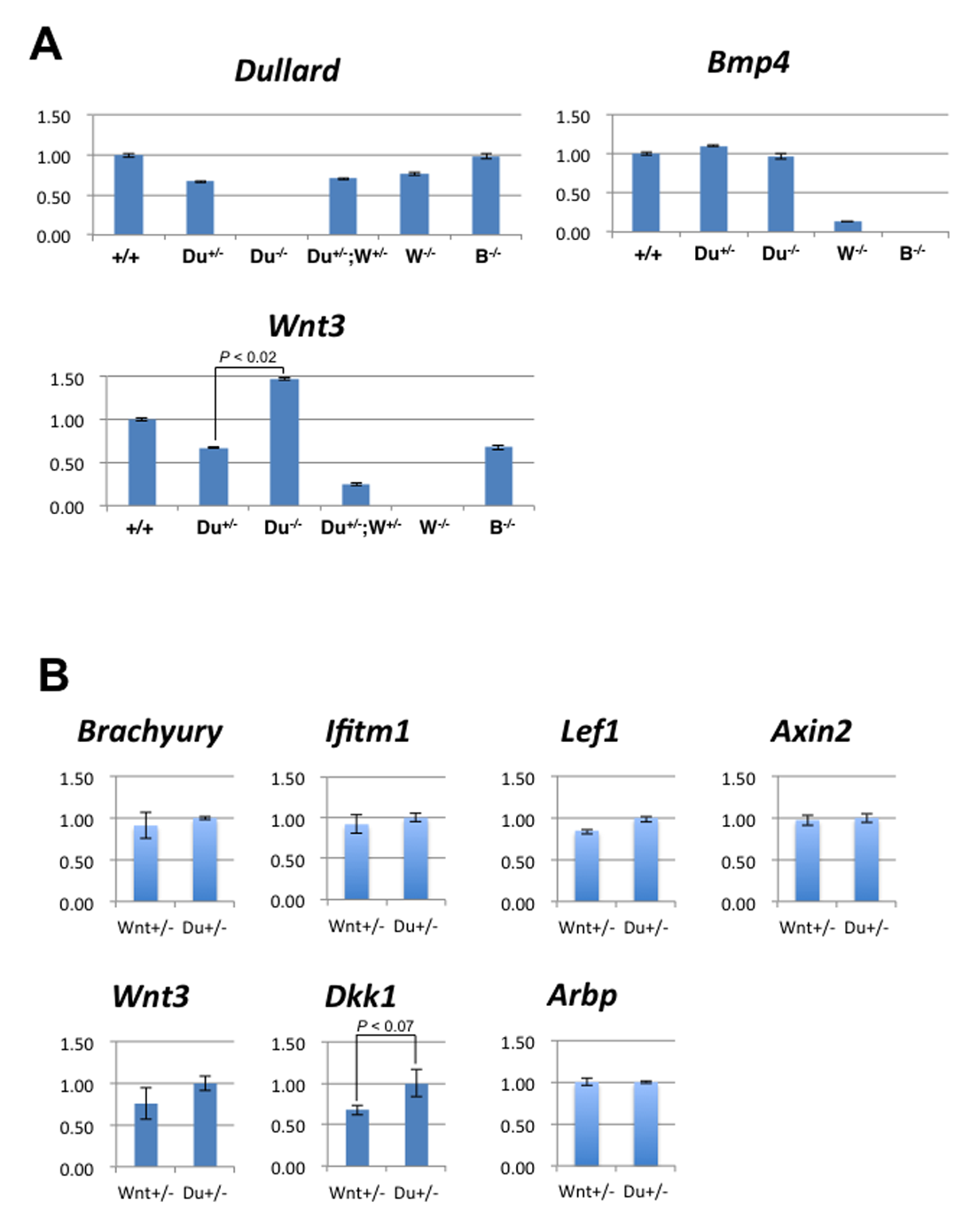

Supplement: Figure S2 — Dullard expression is unaffected by loss of BMP4 or WNT3 functions. (A) Q-PCR analyses of E7.5 Dullard-, Bmp4- and Wnt3-mutant embryos revealed that the expression levels of Dullard in Wnt3−/− and Dullard +/−; Wnt3+/− embryos were similar to those in the Dullard +/− embryo. The Bmp4−/− embryo expressed Dullard at a level similar to that in the wild-type embryo. Wnt3 was significantly up-regulated in the Dullard−/− embryo (P<0.02, Student’s t test). Bmp4−/− embryos showed similar Wnt3 expression levels as that in the Dullard +/− embryo. The expression level of Bmp4 was unchanged in Dullard +/− and Dullard−/− embryos. mRNA expression levels were normalized to those of Gapdh (internal control), and the levels in wild-type (control) embryos were set to 1. Arbp, internal control. (B) Wnt3 +/− and Dullard+/− embryos showed similar WNT/β-catenin dependent downstream activity. Expression of Brachyury, Ifitm1, Axin2 and Lef1 were not significantly different (P>0.1) between E7.5 Wnt3 +/− (Wnt+/−) and Dullard+/− (Du+/−) embryos. Wnt3 expression in Wnt+/− embryos was about 75% of that in Du+/− embryos (P>0.1). Dkk1 expression in Wnt+/− embryos was reduced to about 68% of that in Du+/− embryos (P<0.07, n = 3 independent embryos). mRNA expression levels were normalized to those of Gapdh (internal control), and the levels in Du+/− embryos were set to 1. Arbp, internal control. (TIF) [file pone.0057428.s002.tif]

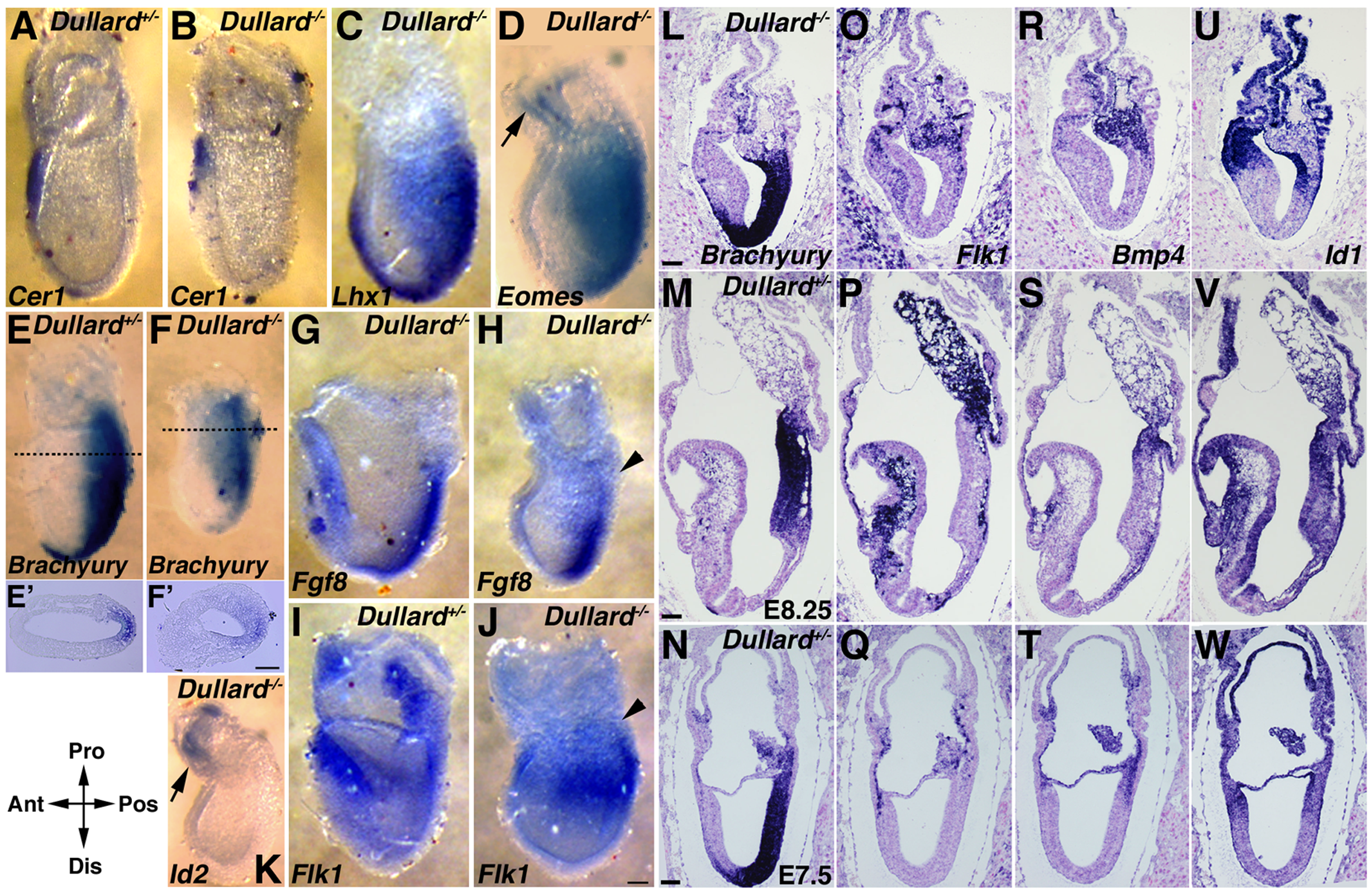

Supplement: Figure S3 — Loss of Dullard function alters the expression domain of germ layer marker genes but does not affect embryonic patterning or the formation of mesoderm. Compared with Dullard +/− embryos, Dullard−/− embryos showed a similar expression pattern of (A, B) Cer1 in the anterior visceral endoderm at E6.5. (C) Lhx1 expression in the posterior and anterior axial mesoderm of the mid streak-stage embryo. (D) Eomes expression in the mesoderm (and also in the extraembryonic ectoderm, arrowhead) of the mid streak-stage embryo. (E, F) Brachyury expression in the primitive streak and nascent mesoderm (but no anterior extension of the expression domain) of the late streak-stage embryo. (E′, F′: transverse sections showing weaker expression in the primitive streak of the null mutant). (G, H) In the late bud-stage embryo, Fgf8 was expressed strongly in the anterior primitive streak but weakly in the posterior segment (arrowhead). (I, J) Flk1 was expressed in the embryonic mesoderm but absent in the extraembryonic mesoderm (arrowhead) of the E7.75 head fold-stage embryo. (K) Id2 was expressed in the extraembryonic ectoderm (arrow) of the E7.5 Dullard−/− embryo, where Eomes was also expressed (see D, arrow). (L–W) Dullard−/− embryos failed to form the allantois and amnion, and showed accumulation of Flk1-, Bmp4- and Id1-expressing cells in their place. In situ hybridization on sections of Dullard−/− embryos for Brachyury (L–N), Flk1 (O–Q), Bmp4 (R–T) and Id1 (U–W). (L, O, R, U) E8.25 Dullard−/− embryos; (M, P, S, V) E8.25 Dullard +/− littermates and (N, Q, T, W) E7.5 Dullard +/− embryos. Pro-Dis, proximal–distal axis; Ant-Pos, anterior–posterior axis. Scale bars = 100 µm. (TIF) [file pone.0057428.s003.tif]

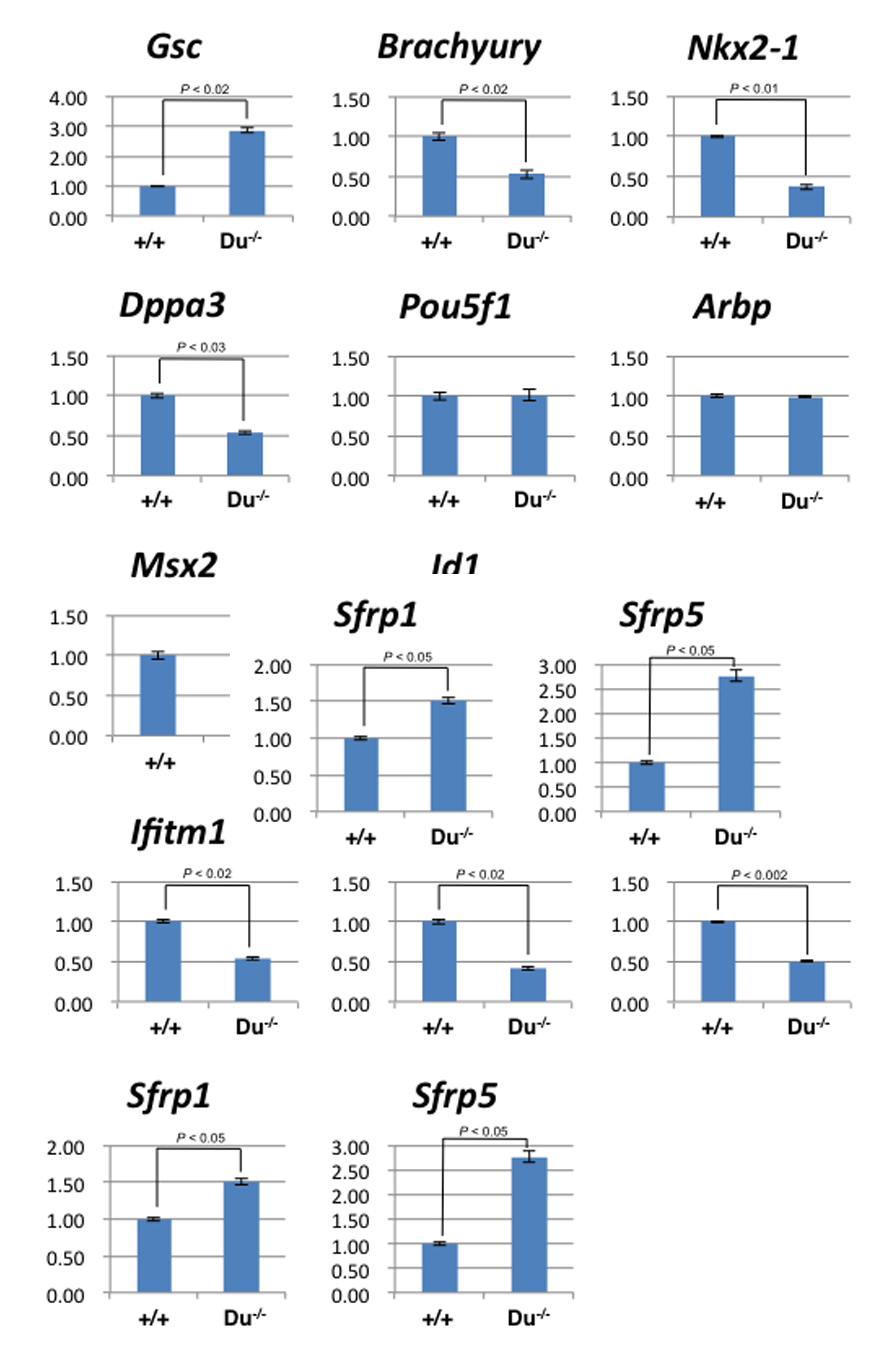

Supplement: Figure S4 — Expression of mesoderm, PGC-related and WNT antagonist genes in E7.5 Dullard−/− embryos. Q-PCR analyses of E7.5 embryos revealed that Gsc (P<0.02, Student’s t-test), Sfrp1 (P<0.05) and Sfrp5 (P<0.05) were up-regulated, whereas Brachyury (P<0.02), Nkx2-1 (P<0.01), Ifitm1 (P<0.02), Lef1 (P<0.02), Axin2 (P<0.002) and Dppa3 (P<0.03) were down-regulated in Dullard−/− embryos (Du−/−), compared with that in wild-type embryos (+/+) (n = 3 independent embryos). Id1 and Msx2 (BMP downstream target genes), Pou5f1 (stem cell marker gene) and Arbp (internal control) expression was unchanged. mRNA expression levels were normalized to those of Gapdh (internal control), and the levels in wild-type (control) were set to 1. (TIF) [file pone.0057428.s004.tif]

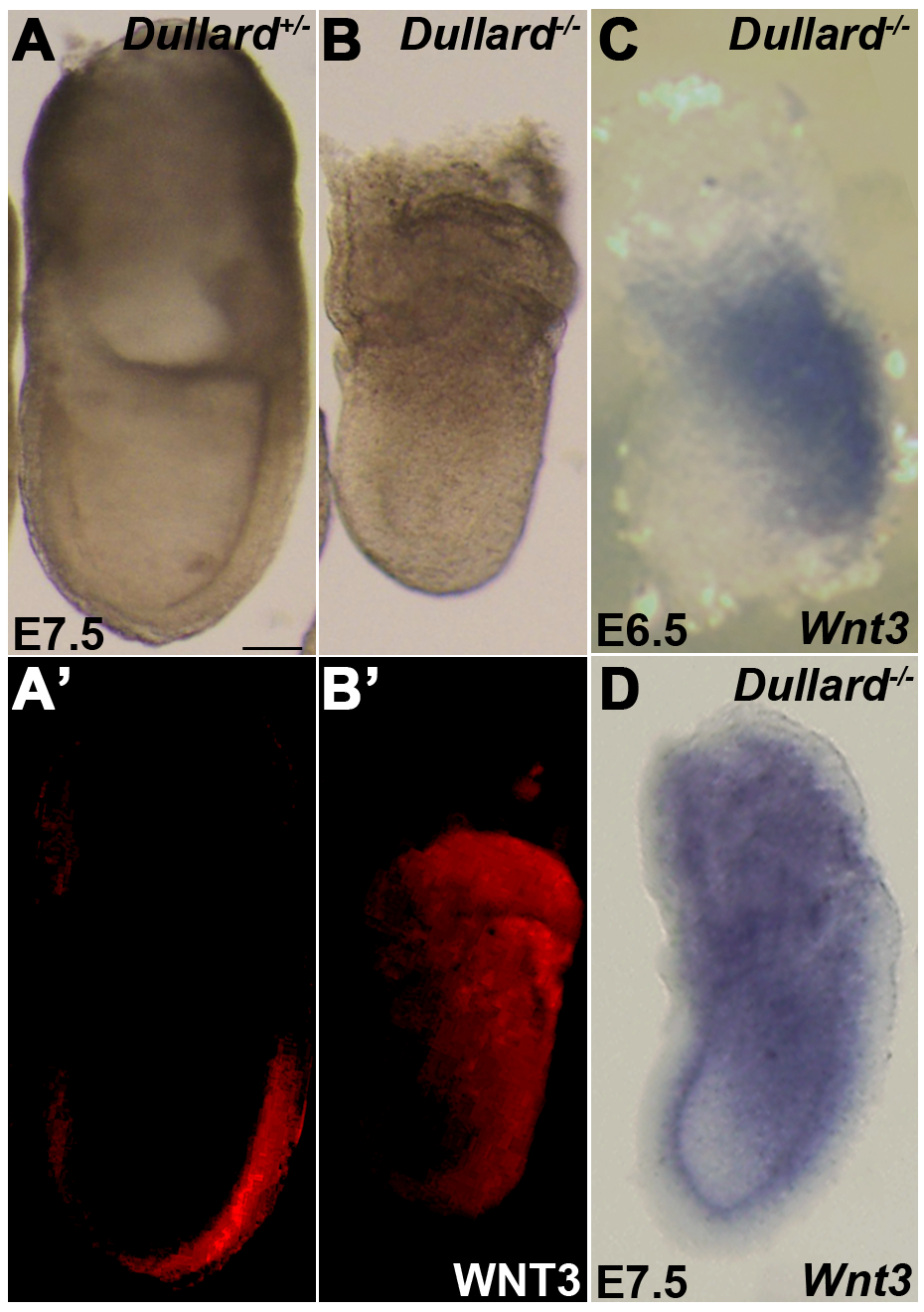

Supplement: Figure S5 — Altered Wnt3 /WNT3 expression in Dullard –/– embryos. Immunofluorescence analysis revealed (A, A′) the regionalized expression of WNT3 (and WNT3A) in the anterior two-thirds of the primitive streak of E7.5 Dullard +/– embryos. (B, B′) Expanded domain of WNT3 (and WNT3A) expression in the posterior germ layers of the E7.5 Dullard –/– embryo. (A, B) Bright-field images; (A′, B′) immunostaining with an anti-WNT3/3A antibody. (C, D) In situ hybridization analysis showing the broad domain of Wnt3 expression in the posterior germ layers of (C) E6.5 and (D) E7.5 Dullard –/– embryos. Scale bars = 100 μm. (TIF) [file pone.0057428.s005.tif]
