# Supplementary material for: Dullard/Ctdnep1 Modulates WNT Signalling Activity for the Formation of Primordial Germ Cells in the Mouse Embryo
Source: PLoS One. 2013 Mar 4;8(3):e57428. doi: 10.1371/journal.pone.0057428 (PMC3587611; doi:10.1371/journal.pone.0057428)
Supplement: Table S1 — Microarray analysis of the expression of genes related to BMP signalling, WNT signalling and PGC formation in E7.5 Dullard+/− embryos versus Dullard+/− embryos. (DOCX) [file pone.0057428.s006.docx]

**Table S1. Microarray analysis of the expression of genes related to BMP signalling, WNT signalling and PGC formation in E7.5 *Dullard^+/–^* embryos versus *Dullard^+/–^* embryos.**

| Gene symbol | Gene name | Fold Change (*Dullard^-/-^* /*Dullard^+/-^*) |
| --- | --- | --- |
| ***Ligands*** | | |
| *Bmp8a* | bone morphogenetic protein 8a | 1.0 |
| *Bmp8b* | bone morphogenetic protein 8b | 1.0 |
| *Bmp5* | bone morphogenetic protein 5 | 1.0 |
| *Bmp6* | bone morphogenetic protein 6 | 0.9 |
| *Bmp7* | bone morphogenetic protein 7 | 0.9 |
| *Bmp2* | bone morphogenetic protein 2 | 0.9 |
| *Bmp4* | bone morphogenetic protein 4 | 0.8 |
| *Wnt10b* | wingless related MMTV integration site 10b | 1.7 |
| *Wnt3* | wingless related MMTV integration site 3 | 1.6 |
| *Wnt4* | wingless related MMTV integration site 4 | 1.2 |
| *Wnt1* | wingless related MMTV integration site 1 | 1.1 |
| *Wnt2b* | wingless related MMTV integration site2b | 1.1 |
| *Wnt6* | wingless related MMTV integration site 6 | 1.1 |
| *Wnt8A* | wingless related MMTV integration site8A | 1.1 |
| *Wnt5a* | wingless related MMTV integration site 5A | 1.0 |
| *Wnt7B* | wingless related MMTV integration site 7B | 1.0 |
| *Wnt9b* | wingless related MMTV integration site 9B | 1.0 |
| *Wnt11* | wingless related MMTV integration site 11 | 0.9 |
| *Wnt16* | wingless related MMTV integration site 16 | 0.9 |
| *Wnt7A* | wingless related MMTV integration site7A | 0.8 |
| *Wnt8b* | wingless related MMTV integration site8b | 0.7 |
| *Wnt3A* | wingless related MMTV integration site 3A | 0.5 |
| *Wnt10a* | wingless related MMTV integration site 10a | 0.5 |
| ***Antagonists*** | | |
| *Nog* | noggin | 1.0 |
| *Chrd* | chordin | 1.0 |
| *Dkk1* | dickkopf homolog 1 | 1.3 |
| *Sfrp5* | secreted frizzled-related protein 5 | 2.0 |
| *Sfrp1* | secreted frizzled-related protein 1 | 1.5 |
| *Sfrp4* | secreted frizzled-related protein 4 | 1.1 |
| *Sfrp2* | secreted frizzled-related protein 2 | 1.0 |
| *Frzb* | frizzled-related protein | 1.0 |
| ***Recptors*** | | |
| *Bmpr1b* | bone morphogenetic protein receptor, type 1B | 1.3 |
| *Bmpr2* | bone morphogenic protein receptor, type II | 1.2 |
| *Bmpr1a* | bone morphogenetic protein receptor, type 1A | 1.0 |
| *Lrp5* | low density lipoprotein receptor-related protein 5 | 1.0 |
| *Lrp6* | low density lipoprotein receptor-related protein 6 | 1.0 |
| *Fzd5* | frizzled homolog 5 | 1.6 |
| *Fzd7* | frizzled homolog 7 | 1.6 |
| *Fzd8* | frizzled homolog 8 | 1.6 |
| *Fzd6* | frizzled homolog 6 | 1.3 |
| *Fzd1* | frizzled homolog 1 | 1.0 |
| *Fzd2* | frizzled homolog 2 | 1.0 |
| *Fzd4* | frizzled homolog 4 | 0.9 |
| *Fzd3* | frizzled homolog 3 | 0.8 |
| *Fzd10* | frizzled homolog 3 | 0.8 |
| *Fzd9* | frizzled homolog 9 | 0.7 |
| ***Signal transducers*** | | |
| *Smad1* | MAD homolog 1 | 1.3 |
| *Smad4* | MAD homolog 4 | 1.1 |
| *Smad5* | MAD homolog 5 | 1.1 |
| *Smad8* | MAD homolog 8 | ND |
| *Ctnnb1* | catenin (cadherin associated protein), beta 1 | 1.3 |
| *Dvl2* | dishevelled 2, dsh homolog | 1.1 |
| *Dvl3* | dishevelled 3, dsh homolog | 1.0 |
| *Dvl1* | dishevelled 1, dsh homolog | 0.9 |
| ***Downstream targets and mesmoderm related genes*** | | |
| *Gsc* | goosecoid homeobox | 6.9 |
| *Lefty1* | left right determination factor 1 | 4.5 |
| *Nodal* | nodal | 2.0 |
| *Msx2* | homeobox, msh-like 2 | 1.2 |
| *Id1* | inhibitor of DNA binding 1 | 1.0 |
| *Axin2* | axin2 | 0.9 |
| *Ifitm1* | interferon induced transmembrane protein 1 | 0.9 |
| *Lef1* | lymphoid enhancer binding factor 1 | 0.7 |
| *Nkx1-2* | NK1 transcription factor related, locus 2 | 0.6 |
| *T* | brachyury | 0.4 |
| *Fgf4* | fibroblast growth factor 4 | 0.3 |
| ***Germ cell/ progenitor markers*** | | |
| *Pou5f1* | POU domain, class 5, transcription factor 1 | 1.0 |
| *Prdm1* | PR domain containing 1, with ZNF domain | 1.0 |
| *Ifitm3* | interferon induced transmembrane protein 3 | 0.9 |
| *Six4* | sine oculis-related homeobox 4 homolog | 0.8 |
| *Dppa3* | developmental pluripotency-associated 3 | 0.4 |
